# Clinical Review and Hospital Reports

**Published:** 1835-07-01

**Authors:** 


### Clinical Review.

### Birmingham Infirmary.

### Reports of the Out-Cases during the Year 1834.*

These reports are two in number—one drawn up by Mr. Parsons, and referring to the cases attended by him; the other by Mr. Ryland, and drawn from his cases. They are chiefly constructed for statistical purposes, and in that point of view are not devoid of interest.

It appears that Mr. Parsons attended, during the year 1834, 3342 cases, of which 132 proved fatal. The three following tables shew the number of cases and of deaths which occurred in each month, distinguishing those under the age of ten years from those above that age, and distinguishing, also, the males and females.

| | MALES. | | | | FEMALES. | | | |
|---|---|---|---|---|---|---|---|---|
| | Under 10 years. | | Above that age. | | Under 10 years. | | Above that age. | |
| | CASES. | DIED. | CASES. | DIED. | CASES. | DIED. | CASES. | DIED. |
| January .... | 43 | 3 | 95 | 5 | 45 | 3 | 152 | 5 |
| February ... | 35 | 1 | 92 | 7 | 41 | 0 | 145 | 3 |
| March...... | 45 | 1 | 70 | 2 | 38 | 2 | 115 | 5 |
| April ...... | 29 | 2 | 80 | 5 | 43 | 4 | 140 | 3 |
| May........ | 43 | 5 | 73 | 1 | 40 | 2 | 117 | 0 |
| June ....... | 35 | 2 | 83 | 6 | 40 | 2 | 111 | 0 |
| July........ | 27 | 1 | 77 | 3 | 29 | 1 | 123 | 1 |
| August .... | 37 | 2 | 62 | 3 | 39 | 3 | 144 | 3 |
| September .. | 47 | 5 | 74 | 6 | 37 | 5 | 110 | 3 |
| October .... | 38 | 3 | 61 | 6 | 32 | 1 | 139 | 2 |
| November .. | 32 | 1 | 81 | 1 | 44 | 1 | 108 | 5 |
| December .. | 33 | 2 | 71 | 2 | 29 | 0 | 118 | 3 |
| Total.... | 444 | 28 | 919 | 47 | 457 | 24 | 1522 | 33 |

| | TOTAL MALES. | | TOTAL FEMALES. | | OF BOTH SEXES. | |
|---|---|---|---|---|---|---|
| | CASES. | DIED. | CASES. | DIED. | CASES. | DIED. |
| January ... | 138 | 8 | 197 | 8 | 335 | 16 |
| February .. | 127 | 8 | 186 | 3 | 313 | 11 |
| March .... | 115 | 3 | 153 | 7 | 268 | 10 |
| April...... | 109 | 7 | 183 | 7 | 292 | 14 |
| May .. ... | 116 | 6 | 157 | 2 | 273 | 8 |
| June ...... | 118 | 8 | 151 | 2 | 269 | 10 |
| July ...... | 104 | 4 | 152 | 2 | 256 | 6 |
| August .... | 99 | 5 | 183 | 6 | 282 | 11 |
| September.. | 121 | 11 | 147 | 8 | 268 | 19 |
| October ... | 99 | 9 | 171 | 3 | 270 | 12 |
| November.. | 113 | 2 | 152 | 6 | 265 | 8 |
| December.. | 104 | 4 | 147 | 3 | 251 | 7 |
| Total.... | 1363 | 75 | 1979 | 57 | 3342 | 132 |

* Prov. Transactions, vol. iii.

"In the next table, the 3342 cases are arranged according to their occurrence before or after the age of ten years, and in the one or the other sex, but without reference to the period of the year; the proportionate mortality is likewise given in each instance. I have also added another column, headed thus, 'For 1833 and 1834,' in which is given the proportionate mortality of the cases which occurred in both these years.

| | Under 10 years. | Died. | Proportionate mortality. | For 1833 and 1834. |
|---|---|---|---|---|
| Males . . . . . . . . . | 444 .. | 28 | 1 to 15,85 | 1 to 14,51 |
| Females . . . . . . . . | 457 .. | 24 | 1 to 19,04 | 1 to 13,84 |
| | 901 | 52 | 1 to 17,33 | 1 to 14,2 |
| | Above that age. | | | |
| Males . . . . . . . . . . | 919 .. | 47 | 1 to 19,55 | 1 to 26,36 |
| Females . . . . . . . . | 1522 .. | 33 | 1 to 46,12 | 1 to 41,45 |
| | 2441 | 80 | 1 to 30,5 | 1 to 34,32 |
| | Males. | | | |
| Under 10 yeais .. | 444 .. | 28 | 1 to 15,85 | 1 to 14,58 |
| Above that age . . . | 919 .. | 47 | 1 to 19,55 | 1 to 26,36 |
| | 1363 | 75 | 1 to 18,17 | 1 to 20,84 |
| | Females. | | | |
| Under 10 years .. | 457 .. | 24 | 1 to 19,04 | 1 to 13,84 |
| Above that age . . . | 1522 .. | 33 | 1 to 46,12 | 1 to 41,45 |
| | 1979 | 57 | 1 to 34,72 | 1 to 29,14 |
| | Total | | | |
| Males . . . . . . . . . . | 1363 .. | 75 | 1 to 18,17 | 1 to 20,84 |
| Females . . . . . . . . | 1979 .. | 57 | 1 to 34,72 | 1 to 29,14 |
| | 3342 | 132 | 1 to 25,32 | 1 to 25,11" |

From the first table it would appear, what has been formerly, we believe, observed, that more males than females die; and that January and September, the depth of Winter, in fact, and Autumn, are the months most fatal to human life. Were the average of the mortality for the years 1833, 1834, to be assumed as nearly representing the truth, with respect to the value of male and female life, there would be this enormous difference in that of the respective sexes—that one male dies out of every 26, above the age of ten years, whilst only one female out of 41 is carried off. Probably this great disparity in Birmingham is accounted for, in some degree, by the unhealthy or laborious occupations of the artisans. The mortality, in both sexes, is proportionately greater below the age of ten years than above it.

The two next tables exhibit the frequency, and the proportionate mortality, of chronic bronchitis and of phthisis.

"CHRONIC BRONCHITIS.

| | MALES. | | FEMALES. | |
|---|---|---|---|---|
| 1st Quarter.... | 33 | 7 died. | 48 | 1 died. |
| 2d Quarter.... | 18 | 0 died. | 22 | 1 died. |
| 3rd Quarter.... | 12 | 0 died. | 12 | 1 died. |
| 4th Quarter.... | 22 | 2 died. | 35 | 2 died. |
| | 85 | 9 died. | 117 | 5 died. |

| | | For 1833 and 1834. |
|---|---|---|
| The 202 cases of chronic bronchitis are, to the whole number of cases of disease, as . . | 1 to 16,55 .. | 1 to 1714, |
| The deaths, 14, are, to the 202 cases, as . . | 1 to 14,43 .. | 1 to 15,54 |
| ——— to the whole number of deaths, as . . . . . . . . . . . . | 1 to 9,43 .. | 1 to 10,61 |
| ——— to the whole number of cases of disease, as . . . . . . . . . | 1 to 238,71 .. | 1 to 266,36 |

In the following two lines I have given the proportion which the deaths from consumption alone bear to the whole number of deaths, and the whole number of cases, which occurred in the two years 1833 and 1834.

| | |
|---|---|
| The proportionate mortality for the whole number of deaths is, as . . . . . . . . . . . . . . . . . . . | 1 to 4,64 |
| of cases of disease is, as . . . | 1 to 116,53." |

The other tables which we shall introduce relate to fever, rubeola, scarlatina, variola, and hooping-cough.

"SYNOCHUS AND TYPHUS.

| | MALES. | | | | FEMALES. | | | |
|---|---|---|---|---|---|---|---|---|
| | Under 10 years. | | Above. | | Under 10 years. | | Above. | |
| 1st Quarter.. | 17 | 0 died. | 14 | 1 died. | 29 | 0 died. | 22 | 0 died. |
| 2d Quarter.. | 11 | 1 died. | 9 | 1 died. | 10 | 0 died. | 4 | 0 died. |
| 3rd Quarter.. | 13 | 1 died. | 11 | 2 died. | 9 | 2 died. | 21 | 1 died. |
| 4th Quarter.. | 25 | 3 died. | 24 | 3 died. | 40 | 2 died. | 42 | 3 died. |
| | 66 | 5 died. | 58 | 7 died. | 88 | 4 died. | 89 | 4 died. |

| | | For 1833 and 1834. |
|---|---|---|
| The 301 cases of fever are, to the whole number of cases of disease, as . . . . . . . | 1 to 11,1 .. | 1 to 14,51 |
| The deaths, 20, are, to the 301 cases, as . . | 1 to 15,05 .. | 1 to 15,84 |
| ——— to the whole number of deaths, as . . . . . . . . . . . . | 1 to 6,6 .. | 1 to 9,28 |
| ——— to the whole number of cases of disease, as . . . . . . . . . | 1 to 16,71 .. | 1 to 22,994 |

RUBEOLA.

| | MALES. | | FEMALES. | |
|---|---|---|---|---|
| 1st Quarter.. | 1 | 0 died. | 7 | 0 died. |
| 2nd Quarter.. | 23 | 4 died. | 28 | 3 died. |
| 3rd Quarter.. | 10 | 1 died. | 10 | 1 died. |
| 4th Quarter.. | 5 | 0 died. | 0 | 0 died. |
| | 39 | 5 died. | 45 | 4 died. |

All these cases occurred under the age of ten years.

| | | |
|---|---|---|
| The 84 cases of measles are, to the whole number of cases of disease, as . . . . . . . . . . . . . . . . . . . | 1 to | 39,78 |
| The deaths, 9, are, to the 84 cases, as . . . . . . . . . . | 1 to | 9,33 |
| ——— to the whole number of deaths, as . . . | 1 to | 14,67 |
| ——— to the whole number of cases of disease, as | 1 to | 371,33 |

### SCARLATINA.

| | MALES. | | | | FEMALES. | | | |
|---|---|---|---|---|---|---|---|---|
| | Under 10 years. | | Above. | | Under 10 years. | | Above. | |
| 1st Quarter.. | 0 | 0 died. | 1 | 0 died. | 0 | 0 died. | 2 | 0 died. |
| 2d Quarter.. | 9 | 1 died. | 1 | 0 died. | 8 | 0 died. | 1 | 0 died. |
| 3rd Quarter.. | 8 | 2 died. | 3 | 0 died. | 12 | 1 died. | 3 | 0 died. |
| 4th Quarter.. | 6 | 0 died. | 1 | 0 died. | 2 | 0 died. | 1 | 0 died. |
| | 23 | 3 died. | 6 | 0 died. | 22 | 1 died. | 7 | 0 died. |

| | | | | | For 1833 and 1834. |
|---|---|---|---|---|---|
| The 58 cases of scarlatina are, to the whole number of cases of disease, as . . . . | 1 to | 57,62 | .. | 1 to | 55,32 |
| The deaths, 4, are, to the 58 cases, as . . | 1 to | 14,5 | .. | 1 to | 19, |
| ——— to the whole number of deaths, as . . . . . . . . . . . . | 1 to | 33, | .. | 1 to | 42,34 |
| ——— to the whole number of cases of disease, as . . . . . . . . . . | 1 to | 835,5 | .. | 1 to | 1051, |

### VARIOLA.

| | MALES. | | | | FEMALES. | | | |
|---|---|---|---|---|---|---|---|---|
| | Under 10 years. | | Above. | | Under 10 years. | | Above. | |
| 1st Quarter.. | 10 | 1 died. | 2 | 0 died. | 2 | 1 died. | 0 | 0 died. |
| 2d Quarter.. | 0 | 0 died. | 0 | 0 died. | 1 | 0 died. | 0 | 0 died. |
| 3rd Quarter.. | 1 | 0 died. | 0 | 0 died. | 0 | 0 died. | 1 | 0 died. |
| | 11 | 1 died. | 2 | 0 died. | 3 | 1 died. | 1 | 0 died. |

| | | |
|---|---|---|
| The 17 cases of small-pox are, to the whole number of cases of disease, as . . . . . . . . . . . . . . . . . | 1 to | 196,59." |

Mr. Parsons presents the brief particulars of four cases of variola after vaccination. In one alone would the disease appear to have been severe; all the patients recovered.

### " PERTUSSIS.

| | MALES. | | | | FEMALES. | | | |
|---|---|---|---|---|---|---|---|---|
| | Under 10 years. | | Above. | | Under 10 years. | | Above. | |
| 1st Quarter.. | 13 | 1 died. | 1 | 0 died. | 13 | 2 died. | 1 | 0 died. |
| 2d Quarter.. | 7 | 1 died. | 1 | 0 died. | 9 | 2 died. | 0 | 0 died. |
| 4th Quarter.. | 1 | 0 died. | 0 | 0 died. | 1 | 0 died. | 0 | 0 died. |
| | 21 | 2 died. | 2 | 0 died. | 23 | 4 died. | 1 | 0 died. |

| | | | | | For 1833 and 1834. |
|---|---|---|---|---|---|
| The 47 cases of hooping-cough are, to the whole number of cases of disease, as . . . | 1 to | 71,1 | .. | 1 to | 51,82 |

| | | |
|---|---|---|
| The deaths, 6, are, to the 47 cases, as . . | 1 to 7,83 .. | 1 to 11,83 |
| ———— to the whole number of deaths, as . . . . . . . . . . . | 1 to 22, .. | 1 to 24,75 |
| ———— to the whole number of cases of disease, as . . . . . . . . . | 1 to 557, .. | 1 to 613,17." |

Tables of the cases of constipation, cholera biliosa, diarrhœa, and dyspepsia are added by our author. These, however, possess comparatively little interest. We shall therefore pass them by. But two of the cases of constipation ended fatally. In neither was there any hernia, or sign of internal strangulation. The two patients were men advanced in life, one being 77, and the other 84 years old; they had been bed-ridden for a long time; they suffered very little pain; and their bowels appeared quite insensible to the stimulus of drastic purgatives, and of the injection, per anum, of large quantities of warm water. Permission to examine the bodies was refused.

---

We now turn to the shorter report of Mr. Ryland. The total number of cases attended by him is 2721; of these, 1127 were males, and 1594 females—696 of the latter being married, and 177 widows. The total number of deaths is 126; of these, 65 were males, and 61 females. The following table shows the relative mortality of the two sexes at different ages.

| | Under 1 Year. | Between 1 & 2. | Between 2 & 10. | Between 10 & 20. | Between 20 & 30. | Between 30 & 40. | Between 40 & 50. | Between 50 & 60. | Between 60 & 70. | Between 70 & 80. | Above 80. |
|---|---|---|---|---|---|---|---|---|---|---|---|
| Males . . . . . . | 4 | 6 | 18 | 2 | 4 | 8 | 3 | 9 | 3 | 6 | 1 |
| Females . . . . | 7 | 4 | 17 | 3 | 7 | 5 | 7 | 3 | 6 | 3 | 0 |
| Both sexes . . . . | 11 | 10 | 35 | 5 | 11 | 13 | 10 | 12 | 9 | 9 | 1 |

The number of deaths in both sexes, below the age of ten years, was equal, and above that age it was nearly so, a result not dissimilar to that obtained by Mr. Parsons. The aggregate of the deaths, below ten years of age, constitutes 4-9ths of the total number.

The number of deaths by fever was 13, five being males and eight females. The following table exhibits their relative ages:

| | Under 10. | Between 10 & 20. | Between 30 & 40. | Between 60 & 70. | Total. |
|---|---|---|---|---|---|
| Males . . . . . . . | 2 | 2 | 1 | 0 | 5 |
| Females . . . . . . . | 5 | 2 | 1 | 1 | 8 |
| Both sexes . . . . . . | 7 | 4 | 2 | 1 | 13 |

Fever was very prevalent at Birmingham in the Summer quarter. It chiefly affected the bowels, and occasioned ulceration; for in all the cases examined after death by Mr. Ryland, ulcers were found in the ileum and cæcum.

" One of the cases, in which ulceration of the bowels was found, is curious, from the circumstance of the patient dying of hæmorrhage that had its source in the ulcers. John Taylor, æt. 34, shoemaker, was seized with fever on the 4th of November. Under the usual treatment he seemed to be improving rapidly, and began to take nourishment, when in the night of the 16th, he had two large motions, composed almost entirely of blood."

We lately saw a case of fever, attended with the usual symptoms of ulceration of the mucous membrane of the bowels, in which very frequent hæmorrhage occurred. The patient died, but no distinct ulceration was detected, though the mucous membrane appeared abraded in several parts of the lower extremity of the ileum.

The next table shews the relative mortality of the consumptive patients, with regard to age and sex :

| | Between 20 & 30. | Between 30 & 40. | Between 40 & 50. | Between 50 & 60. | Total. |
|---|---|---|---|---|---|
| Males . . . . . . . | 2 | 7 | 3 | 6 | 18 |
| Females . . . . . . . | 5 | 2 | 3 | 0 | 10 |
| Both sexes . . . . . . | 7 | 9 | 6 | 6 | 28 |

One case of death by small-pox after vaccination is recorded. The child, a boy, æt. 5, was vaccinated on the 4th of August, and the vesicle was large and perfect on the 11th. In the evening of that day he became unwell, and small-pox appeared on the 14th. The boy died convulsed on the 20th.

" Perhaps I may here mention a case of absence of the uterus, as it is of rare occurrence. Hannah Mack, æt. 19, servant, was admitted into the infirmary, on the 29th of August, 1834, with peritonitis, of which she died in thirty-six hours. On examining the body, the peritonitis was found to have arisen from the escape of fæcal matter through an ulcer of the ileon. As the girl had never menstruated, I wished to examine the uterine organs, but no uterus could be found. Within the fold of the broad ligament, where the uterus should have been, there was a firm, round, fibrous cord, four inches long and thicker at the extremities than in the middle. To each extremity of this cord, the fallopian tube of the corresponding side was attached, becoming impervious just previous to its junction with it. The ovaries were of the natural size, the fallopian tubes well developed. There was no trace of a vagina; the external parts below the meatus urinarius were closed by a strong membrane. The body was small, but well formed; the feminine character of the skeleton was marked; there was an unusual quantity of hair on the pubes and in the axillæ; and the mammæ were small, but decidedly formed."

It is not the uterus which would seem to confer the sexual character upon the frame; the ovary appears the important organ. The uterus is merely an organ of relation, a receptacle for the impregnated ovum. The ovarium is to the female, what the testicle is to the male, the centre of the sexual appetite and sexual influence. It would be curious to learn, if that were possible, if this female exhibited any sexual propensities.

In an appendix to the preceding report, Mr. Ryland presents a table, constructed for the purpose of exhibiting the liability of persons, of various ages and of different sexes, to suffer from certain diseases. The females seemed to suffer in a larger proportion than the males; but, as Mr. Ryland justly observes, the greater prevalence of sick clubs for the men may affect the ratio in a material degree. The following is the table referred to.

Mr. Ryland remarks, that an analysis of the preceding table shews a greater frequency of fever and acute bronchitis, below the age of 20 than above it. But chronic bronchitis and chronic rheumatism increase with the increase of years; and females so affected exceed the males in number, nearly in the ratio of 2 to 1. Such, at least, is the deduction from this table.

Dyspepsia would also appear to afflict females more than males. This, indeed, is borne out by common observation, for, in practice, we find the majority of dyspeptics in the former sex. This, probably, is attributable to their sedentary habits.

"In chronic gastritis, or inflammatory dyspepsia, the proportion of females to that of males is nearly as 5 to 1. The largest number of cases occurred between the ages of 10 and 20. Of the three males, 2 were brass-casters; of the 23 females, all were unmarried, 5 were sempstresses, 5 domestic servants, 3 pearl-button makers; and, in almost every instance, the complaint was accompanied by disorder of the menstrual functions, and in most of the cases by amenorrhœa. Of the cases of this disease that occurred between the ages of 20 and 40, several appeared to be referable to the long continued suckling of their children, in which many of the women had indulged."

In the lower class of life, that class from which Mr. Ryland's report is drawn, the abuse of spirituous liquors by women is notorious. A proprietor of a large London gin temple informed us that, for one man who frequented his establishment, there were two or three women at the least. Such is the constitution of the female sex, that at all ages, and in all ranks, it is too prone to yield to the impulse of passion and the gratification of sense.

"Two of the cases of paralysis were instances of palsy of the extensor muscles of the hand and fingers, causing that peculiar dropping of the hand, which is frequently seen in those persons who work much with lead. Both the sufferers were brass-cock founders; and so prevalent is the affection among this class of workmen, that it is familiarly denominated the cockfounder's disease.

I have been at some little pains to seek out the reason of the occurrence of this complaint amongst artizans of the above description, and there can be no doubt that it is owing to the lead, which forms nearly a fourth part of the compound metal of which the cocks are made. The '*drop*' of the hand never affects the *casters*, although they are exposed to the fumes of the heated metal; it occurs only in those workmen who are engaged in the filing and polishing of the brass cocks. During these processes the article is covered with oil or grease of some description, and in order that the workman may see whether sufficient effect has been produced by his file or his lathe, he rubs off the greasy matter, which is full of small particles of the metal, with the palm of the hand or the wrist, and this is done many times in a minute. It is probable that the presence of the grease materially assists the friction and pressure in forcing the lead through the pores of the skin.

The extensors of the hand and fingers, the supinators of the hand, and the flexors of the thumb, are the only muscles that lose their power. One of the patients referred to in the present report, had a regular attack of lead-colic; but it is worthy of remark, that the brass-cock founders very seldom experience any ill effects from the lead, except the dropping of the hands. I have inquired of many men afflicted with the complaint in question, if they had been subject to dyspeptic disorders, to constipation, or to colic, and I have always received an answer in the negative. In painters the colic is far more common than this paralytic affection of the hands; and those who have laboured under the latter, have generally suffered severely from the former disease at previous periods.

One of the cases of paralysis was of that kind called the shaking palsy, or *tremblement mercuriel;* it occurred in an old woman who had formerly been a gilder. This disease is now become very rare in Birmingham, the present improved mode of gilding having done away with the imminent risk of health and life, which formerly attended this branch of manufacture."

We think the desire evinced and the plan adopted by our authors to increase our statistical information highly laudable. These great generalizations are of vast importance, not only in enabling us to obtain accurate general views, but frequently in assisting us in individual cases. General laws being an aggregate only of particulars, are applicable of course to their elucidation.

This terminates Mr. Ryland's report. With it terminates also the clinical portion of the third volume of the Provincial Transactions. We must say that we deem that clinical department meagre, disproportionate to the expectations of the public and the opportunities of the contributors. The Association numbers in its list of members many surgeons of provincial hospitals. Surely they might offer some facts of value, some opinions of interest. They are too supine. To your tents, O Israel, say we.

---

### SIR P. DUN'S HOSPITAL.

#### I. HYDROPS PERICARDII, &c. &c. By Dr. R. LAW.

J. Campbell, aged 19, naturally strong and temperate, was admitted into the above institution on the 1st of May, with œdema of the legs and feet—fluctuation in the abdomen—liver enlarged—dull sound in the præcordial region—respiration puerile in all the anterior right side, and in the superior third of the left—heart pulsating feebly over a space corresponding with the dull sound—posteriorly, the inferior half of each side of chest gave a dull sound, and afforded no respiration. On changing the position, so as to make the shoulders dependent, the sound became clear where it was before dull. He experiences the *decubitus difficilis,* and breathes easily only when sitting up, or inclined to the left side. He has a dry husky cough, and scanty urine. Some parts of the diagnosis were clear enough; but other parts were not so unequivocal.

" We had now to account for the extensive dull sound in the præcordial region; we concluded this to depend either upon extensive dilatation of the heart, or upon equally extensive effusion into the pericardium. We ought to have remarked before, that the account Campbell gave of himself was, that he had had the influenza a month before we saw him, and not finding himself recovering his strength, and perceiving his legs and feet to swell, he was induced to seek admission into hospital. Up to the period of his being attacked with the influenza, he had enjoyed perfect health. A dilatation of the heart, commensurate with the extent of the dull sound, would have required more time to develope itself, than consisted with the history of this case. Besides the ordinary pathognomonic signs of dilated heart were wanting, viz. extension of sound beyond the præcordial limits, the sound here being strictly confined within the space which yielded the dull sound on percussion; nor had he the characteristic sharp, short, loud first sound, but a weak, feeble action of the heart, evidently more remote from the surface than natural."

Mercury and diuretics were employed. He mended very slowly. As the ascites diminished, they had opportunities of estimating the size of the liver. He progressed slowly till the 11th of the July following, when he was suddenly seized with weakness, depression of spirits, followed by purpura hæmorrhagica, the gums being soft and spongy. Under tonics the purpura disappeared, and the former treatment being recurred to, combined with tonics, he recovered to a greater degree than could have been anticipated. The pleuritic effusion vanished—and by the middle of October he applied for his discharge. The report then was—"No œdema of legs or feet—no ascites or anasarca—no sign of fluid in the pleura—sound still dull in the præcordial region, but without producing any inconvenience—can lie in the horizontal posture, and sleep comfortably—no cough—can walk briskly without oppression of breathing—general health improved."

The foregoing case is very interesting in many points of view. The severe sequelæ of the influenza threatened life for a long time, and were happily removed by mercurials and diuretics, carried on till the mouth was affected. After that, the mercurials were laid aside, and the diuretics were combined with tonics. The author has found a liniment composed of volatile liniment, turpentine, and tincture of cantharides, well rubbed over the abdomen and sides of the chest, an useful auxiliary to the above-mentioned plan of treatment, in removing effused fluid from the cavities.

### II. Effusion into the Pericardium and Pleuræ.

Lætitia Green, aged 13 years, was seized with scarlatina in the hospital, leaving a dry hard cough and dyspnœa, obliging her to sit up in bed. She complained of distressing palpitation—face bloated—lips livid—feet œdematous—pulse 96, and pretty full.

" Percussion anteriorly yielded a clear sound in all the right and in the superior two-thirds of left side: posteriorly the sound is clear in the superior two-thirds, and dull in the inferior third of each side. In the præcordial region, or the anterior inferior left third, there is palpable fulness and dullness of sound. Although the respiration is distinct in all points when the sound is clear, still it has a peculiar short, abrupt character, as if the vesicular structure of the lung could not expand itself, or open for the reception of the air. When the sound posteriorly was dull, respiration was absent; but these phenomena disappeared on change of posture. On placing the patient on her hands and knees, with the body sloping, clearness of sound and distinct respiration took their place. We thus had the assurance, that it was upon fluid effused into the pleuræ they depended, and that this fluid was not prevented by adhesion from obeying the laws of gravitation."

The heart beat strongly, and with a marked "bruit de soufflet"—the bruit being most distinct in the left side. After much reasoning and some doubt, they came to the following diagnosis:—"Effusion into the cavity of each pleura, and into the pericardium, the result of pericarditis." Diuretics, digitalis, &c. were exhibited internally, and the liniment before alluded to was applied to the chest. These were continued from the 25th of May till the 9th of June, when the countenance appeared to be much improved—the swellings of the face and feet gone—decubitus facilis—cough nearly well—pulse 54—fulness still in the præcordial region—" the *bruit musical*, exactly resembling the chirping of a young bird, had taken the place of the *cidevant* " bruit de soufflet."

Tonics were now combined with the diuretics.

"June 16. Pulse 84, full, firm, and regular; bruit musical, very distinct; on examining the posterior region of the chest we heard as distinct bruit de soufflet through all this region, as we had ever heard it under its most striking and unequivocal character in the præcordial region; it prevented us hearing the respiration. It occurred to us to make the patient suspend her breath for a few moments, which when she did, the phenomenon completely disappeared. We directed the attention of several experienced stethoscopists to the case, and after they had made their examination, asked their opinion as to the sound and its cause. All regarded it as an unequivocal bruit de soufflet, dependent upon an affection of the heart. Their surprise was great when they found that stopping the respiration caused it to vanish. We have here an interesting example of the manner in which diagnostic signs which are usually to be depended upon, may sometimes deceive us. Had the respiration retained its physiological relation to the circulation, the error could not have been committed; but in the case in question this relation was lost, and the respiration and pulse were nearly equal in point of number. Our patient amended steadily, and found herself so well on the 10th of July, that she applied for permission to leave the hospital. Report on that day:—no bruit de soufflet, nor modification of it in the præcordial region; fulness in the region quite gone, nor does the dull sound exceed its normal limits; the respiration posteriorly still retains its peculiar resemblance to bruit de soufflet, but is heard much more inferiorly; cough quite gone; no œdema of feet even at night; she can go up stairs without feeling the least distress of breathing."

The termination of the case convinced Dr. Law that effusion into the pericardium, apparently the result of pericarditis, was the cause of the phenomena.

III. Pericarditis from Rheumatism.

In this case the patient had been freely bled, and taken vin. colchici. A translation suddenly took place to the heart.

"July 11th. *Present symptoms*; pulse 90, regular, full and strong; skin hot; respiration hurried, irregular, (*entrecoupée*) and panting; heart's action apparent to the eye; percussion yields a dull sound in the præcordial region, and even beyond it, both transversely and from above downwards; elsewhere the sound is quite clear. The stethoscope, applied to the space marked by the dull sound, recognizes a violent action of the heart, but so tumultuous and confused, that the different sounds of the organ cannot be distinguished."

He was bled to 16 ounces—leeches were applied to the cardiac region—and calomel, opium, antimony, and digitalis were given every three hours. By these means the violence of the complaint was broken—but the recovery was slow, and a distinct bruit de soufflet was left for some time. When this disappeared a strong impulsion remained, to which the patient became accustomed. It is clear, from this and many other cases, that the "bruit de soufflet" is not always indicative of a narrowing of passages through which the blood rushes. It is very probable that it sometimes is produced by pericarditis. There is no doubt also, that violent actions alone of the heart will produce the phenomenon for a short time. Of this we have seen several examples.

IV. Some account is given of a case where the aortic valves were suspected to be defective. The patient was a labourer, 38 years of age, and habitually exposed to atmospheric vicissitudes. His symptoms, when admitted, were dulness of sound in the præcordial region—strong impulse of the heart—bruit de soufflet—pulse 84, full, strong, and vibrating—pulsation of various arteries visible—hard, dry cough—respiration distinct in all parts of the chest, except where disguised by the heart's action—palpitation when lying on the left side, cough when on the right—palpitation comes on every night at ten o'clock, obliging him to sit up—complexion pale and sallow—no œdema—

subject to epistaxis. He was bled to ten ounces, and much relieved by the loss. Camphor, digitalis, and prussic acid were given, under which he so much improved as to be able to leave the hospital; but he soon returned, with the action of the heart so violent as to make the whole bed shake. The dyspnœa was also very distressing. He was again bled, and superacetate of lead with opium was given, together with other medicines. The changes were insignificant, and not noted down always. After a time a strong abdominal pulsation, attended by a well-marked "bruit de soufflet" took place. There was no tumor perceptible, nor tenderness on pressure. They came to the conclusion that these phenomena were of a nervous character, and gave him tonics with antispasmodics and improved diet. Under this plan the abdominal pulsation nearly disappeared —the bruit entirely. He left the hospital—but they learnt that he died in about a month afterwards. There was something more therefore than nerves in the case.

Some remarks are appended by Dr. Law on that curious and puzzling phenomenon—epigastric pulsation; but nothing satisfactory is elicited. For our own parts, we think it always depends on action of the heart, and not of the arteries.

---

### Birmingham Eye Infirmary.

### Annual Report of the Birmingham Infirmary for Diseases of the Eye. By R. Middlemore, Esq.

This report is contained in the third volume of the Transactions of the Provincial Medical and Surgical Association. Its author, Mr. Middlemore, is known as an active and zealous observer of diseases of the eye, and the profession are indebted to him for several valuable contributions on the subject.

The cases that have occurred at the infirmary within the year are as follow: Simple acute conjunctivitis, 236. Chronic conjunctivitis, 126. Acute conjunctivitis, with pustules on the conjunctiva, 105. Acute conjunctivitis, with pustule or ulcer of the cornea, 112. Acute conjunctivitis, with puriform secretion, 75. Purulent conjunctivitis of newly-born infants, 33. Irritable conjunctivitis, 36. Strumous conjunctivitis, 78. Pterygium, 9. Corneitis, 24. Vascularity of the cornea, 10. Opacity of the cornea, 123. Staphyloma of the cornea, 13. Impaction of foreign bodies in the cornea, 23. Simple acute sclerotitis, 5. Rheumatic sclerotitis, 9. Affections of the membrane of the aqueous humor, 11. Simple acute iritis, with or without ulcer or opacity of the cornea, onyx, or hypopium, 59. Chronic iritis, 7. Syphilitic iritis, 3. Strumous iritis, 8. Arthritic iritis, 2. Prolapse of the iris, 7. Fungus from the iris, 2. Cataract, 29. Dislocation of the lens, 11. Choroiditis, 4. Retinitis, 4. Glaucoma, 8. Hydrophthalmia, 1. Suppuration of the eye-ball, 4. Atrophy of the eye-ball, 4. Fungoid, and various anomalous tumors of the eye-ball, 2. Amaurosis of various kinds and degrees, 110. Diseases of the lachrymal caruncle and semilunar membrane, 4. Diseases of the lachrymal passages, 23. Epiphora, 10. Strabismus, 7. Tinea, 91. Lippitudo, 17. Hordeolum, 8. Ectropium, 2. Entropium, 6. Inflammation of the eye-lids, 13. Œdema of the eye-lids, 6, Ptosis, 5. Ulceration of the eye-lids, 8. Adhesion of the eye-lid to the globe, 4. Tumors in the eye-lids, 28. Wounds of the eye-ball and its appendages, 39.

The affections singled out by Mr. Middlemore for notice are development of the cornea—opacity of the cornea —staphyloma—cataract—amaurosis— and, lastly, acute inflammation of the semilunar membrane and lachrymal caruncle.

#### I. *Development of the Cornea.*

"When the cornea is small from birth, it usually happens that the other parts of the eye exist in a correspondently diminished size. This defect I have often witnessed; and since the publication of my last Report, I have also seen two instances in which the cornea was scarcely at all developed, the other parts of the eye being apparently perfectly formed. I have also seen two examples of undue development of the cornea. The first of these examples is thus referred to in my printed Introductory Lecture: 'I have lately seen a young man from Bloxwich, suffering from a most extraordinary development of the left cornea; though retaining the most perfect transparency, it is increased to nearly treble its natural size; the iris has augmented in a corresponding degree, so that the anterior chamber is amazingly ample. The other parts of the eye are not at all enlarged. But the fact to which I am desirous of directing your particular attention, is the occurrence of the same disease in the opposite cornea. When I first saw this person, the right cornea was not at all enlarged, but it is now evidently increasing, and will soon, I fear, become as large as the other, at least, unless something be done to prevent it.'

In the second example, the disease occurred after small-pox, in an infant, a patient of my friend Mr. Palmer, who requested me to see the case. The right eye is quite healthy. The cornea of the left eye is much increased in extent, its diameter being at least twice as great as that of the opposite cornea. Its texture is apparently healthy, and quite transparent; the anterior chamber is very ample, and the iris is placed at a considerable distance from the neural surface of the cornea. The humours of the eye are somewhat cloudy. If the left cornea continues to increase in size, and if a morbidly increased development occur in the opposite cornea, and which the use of iodine and mercury, and the employment of counter-irritation, should fail to arrest, I shall recommend, *from experience of its utility* in such cases, the removal of a central portion of the much enlarged cornea, as a means of preventing the loss of vision in the eye last affected. This is the only mode of destroying the sympathy which exists between these parts under this condition of disease, with which I am acquainted." 374.

II. *Opacity of the Cornea.*

Mr. Middlemore is of opinion that the frequency of the occurrence of opacity of the cornea is a proof either of the neglect of patients, or of inefficient treatment on the part of the practitioner. There are some points connected with the history of corneal opacity, which he thinks have been insufficiently explained. He observes that when the clearness of the cornea is slightly impaired by the existence of chemosis around its margin, the secretion from the cellular web, which connects together its lamellæ, becomes turgid; and if the original malady continue, interlamellar lymphatic deposition takes place, and, presuming that the prior affection is then remedied, the cornea remains opaque; there remains, in fact, a slight form of diffused onyx. After some severe fevers, and in that prostrate condition of the system which is produced by the malignant cholera, or which remains after copious hæmorrhages, a similar state of things has been remarked; and it will be remembered, that it is a preliminary state to the sloughing of the cornea and suppuration of the eye-ball, when the nutrition of the globe has been interfered with by the division of the fifth pair of nerves.

In many instances of this diffused interlamellar deposition, the transparency of the cornea is entirely restored by the adoption of very simple measures, the opaque substance being gradually removed. But if the cause which gave rise to the interlamellar deposition continues in operation, ulceration, staphyloma, or sloughing of the cornea may be looked for, in consequence of the interruption occasioned to the nutrition of the corneal lamellæ.

"If a thin layer of inflammatory (lymphatic) deposition exist between the corneal lamellæ, it will produce a dirty bluish-white appearance, such as is often witnessed in the light form of *opacity of the cornea;* and it is to this condition of disease that the following plan of treatment is more particularly adapted. Let a camel hair pencil be dipped in a solution of the sulphate of cadmium (one grain to two ounces of distilled water) and applied to the cloudy part of the cornea three or four times a day.

This application produces very little uneasiness; and I have not observed that it inflames the eye, or produces any of those unpleasant effects which sometimes follow the use of the oxymuriate drops. I do not usually advise the solution to be used until the cornea is *entirely* removed: and I think it unnecessary to continue its use after indications of its *commencing* disappearance are unequivocally manifest."

When opacity of the cornea results from loss of its lamellar texture, the preceding treatment is of course inert. The insufflation of calomel, &c. recommended by Dupuytren and other French surgeons, has proved in Mr. Middlemore's hands unsuccessful and irritating. A weak solution of the oxymuriate of mercury is a common and a good application, if not too early used or too long continued. The point is to avoid producing inflammation, to which the eye affected is prone.

### III. *Staphyloma.*

" Geo. Rhodes, æt. 2, has a very large globular staphyloma of the right eye, consequent on purulent ophthalmia. The surface of the tumour is round, smooth, and very vascular; the eye-ball is much enlarged, but is not the seat of much pain and irritation. The eye has been tapped on several occasions, and various remedies have been tried, but fruitlessly tried, with the intention of checking its growth.

*Operation.*—I removed a portion of the staphylomatous projection (with Beer's knife and the hooked forceps) rather less in extent than the cornea in its natural state.

*Treatment.*—A roller was passed round the eye after the operation, and the nurse was directed to keep that part of it which covered the eye wet with cold water. The child to have a little of the syrup of poppies.

*Result of the case.*—A good deal of swelling of the lids, and tension of the globe, occurred a few days after the operation; but they soon subsided, and the eye-ball is now reduced to its ordinary size.

I have made a preparation of that portion of diseased cornea I excised, and it very well displays the usual circumstances which constitute the pathology of staphyloma of that texture. The cornea is enormously thickened, and is very vascular; the vessels which lead to its organization pass through it irregularly, without any definite arrangement; some of them shoot through its entire texture; but the major part of them ramify as a dense net-work upon, and immediately beneath, its surface. Its lamellar arrangement is totally destroyed; so that its layers are consolidated and cannot be moved upon each other, as in their normal condition. The iris is firmly adherent to the neural surface of the cornea, it is intimately connected, almost identified, with it, and is torn or separated so as to possess a reticulated appearance, in consequence of the extension of that texture to which it was attached before it became materially increased in extent."

Mr. Middlemore remarks that this was an example of *globular* staphyloma, in which the eye attains a larger size, and gives rise to infinitely less irritation than it does in the *conical* variety. He is firmly convinced that had he adopted the usual mode of removing this large staphyloma by excising a great part of its anterior surface, very severe symptoms would have resulted. The disease is cured, not by the mere diminution produced by, and in correspondence with, the size of the part removed, but by the occurrence of atrophy as a consequence of the operation. The principle, therefore, to be kept in view, in the adoption of our remedial measures, is the production of atrophy without the extensive removal of parts by a surgical operation.

### IV. *Cataract.*

" I shall briefly describe," says Mr. Middlemore, "my usual plan of operating upon children from one to three years old, suffering from complete congenital cataract. I have the pupil fully dilated by the use of a strong solution of hyosciamus; the child's body securely enveloped in a napkin, with the arms fixed to the sides; and the child placed upon a table of a convenient height, with the head slightly raised. The head is firmly fixed by an assistant, by pressing the hands on either side of it. If operating upon the right eye, I raise the upper lid and steady the eye-ball (*not by means of a speculum*) by pressing the index finger upon the temporal, and the middle finger upon the nasal side of it. I then *rapidly* pass a very fine needle* through the cornea, near to its junction with the sclerotica, and simply lacerate the capsule by slightly moving its point a little backwards, and to either side.

The advantages of this mode of operating are neither few nor unimportant. In the first place, it is quite efficacious, quite competent to the removal of the disease, or, at most, only requires to be performed a second time; secondly, when properly performed, it involves no risk of injuring any important texture, except the cornea; thirdly, it gives rise to scarcely any pain; and fourthly, it excites hardly an appreciable amount of inflammation."

Mr. Middlemore denounces the speculum as equally painful and injurious in its operation. He cites the opinions of his professional friends in favour of the method he adopts.

### V. *Amaurosis.*

Mr. Middlemore earnestly entreats his breathen to test the effects of strychnia in amaurosis. With those effects he is amply satisfied, and he appears a warm and experienced advocate of the value of this remedy. Yet he thinks that it has been employed in an indiscriminate manner, and he feels convinced that all who employ it with judgment and with care, will form the same conclusions of its qualities as he has done.

* The needle should be very fine and slender, sharp *at* the point, a little flattened *towards* the point, having a cutting edge on either side for a short distance *from* the point, and gradually becoming round towards the handle.

### VI. *Acute inflammation of the Semilunar Membrane and Lachrymal Caruncle.*

"A girl, about twelve years old, came to the infirmary, with a very considerable enlargement of the semilunar membrane and lachrymal caruncle. The enlarged parts were very red, but scarcely at all painful. She could give no account of the cause and progress of the complaint, which was, however, soon removed by the application of leeches near the inner canthus, and by free and frequently repeated scarification. As this young person was recovering, her sister applied to me with a precisely similar condition of disease, which, it appeared, had existed for about a month; during which time she had, with the approbation of her medical attendant, been under the care of a cobbler of this town, who had put into her eye, every day, *a dark-coloured ointment*, with the assistance of a *knitting-pin*. This case was also relieved by the plan of treatment which was so successfully employed for her sister. But it is somewhat remarkable (it may, perhaps, be a consequence of the improper treatment she underwent, combined with the longer duration of the disease) that the semilunar membrane and lachrymal caruncle remain in a state of enlargement, which, I apprehend, nothing short of a surgical operation will remove."

This concludes the report, with which we have but one fault to find—its brevity.

---

### Glasgow Infirmary.

### Dr. Macfarlane's Report on Tumors of the Abdomen.*

In the clinical department of the last number of this Journal, we presented an account of part of an extended report on tumors by Dr. Macfarlane of Glasgow. The report in question occupies sixty pages of that gentleman's volume of clinical reports. The first

* Clinical Reports, &c. By J. Macfarlane, M.D.

half is devoted to tumors of the head and neck, and tumors of the mamma. That has been already brought before our readers; the last half of the report (about thirty pages), is occupied with observations on, and cases of tumors of the abdomen, and to this we shall now direct attention.

Previously to entering on this interesting subject, we may make a remark with reference to tumors of the mamma. This organ is occasionally affected with morbid alterations in the male. We have seen one instance of scirrhus of the male mamma, and we lately heard of another. A month or two ago, we saw a case rather curious in some of its features. A gentleman who had never, so far as he knew, been affected with primary symptoms, had ulceration of the throat and an eruption, exhibiting all the characters of the syphilitic lepra. Soon after this, he got chronic inflammation of the testis, and soon afterwards ulcerations of the mucous membrane of the rectum. About this time, one mamma became much enlarged, very hard, and extremely painful upon pressure. We put him on a course of calomel and opium, and the morbid enlargement of the breast subsided, along with the syphilitic symptoms.

We have frequently seen chronic inflammation of the testis, in connexion with secondary symptoms, especially with those of a cachectic kind; but we never previously observed an affection of the mamma in such circumstances. But the mamma and the testis may be looked on, we imagine, as organs presenting some analogies of structure, and at all events, the proper glandular tissue is, in both, mixed up with much fibrous texture.

We now revert to—

### Tumors of the Abdomen.

It might appear, à priori, that the diagnosis of tumors of the abdomen would be simpler than that of morbid changes of structure in the thorax, for the soft and yielding parietes of the former might seem well calculated to present facilities of examination, denied by the bony walls of the latter. Yet experience does not confirm this natural expectation. The functions of the heart and lungs are more determinate and distinct, and the means of ascertaining their operations are, thanks to auscultation, more precise than is the case with respect to the viscera of the abdomen. The circumstances that interfere with the appreciation of their disturbances of function and of structure, are well pointed out by Dr. Macfarlane. We draw attention to them, because we have seen so many and such glaring errors committed, in the diagnosis of abdominal tumors, that the necessity of being acquainted with all that is ascertained upon the subject, ought to be made apparent to all who would wish to practise the profession with credit and advantage.

"It is well known (says Dr. Macfarlane) that, in a state of health, certain viscera are contained in certain regions of the abdomen; but, when we consider that many of these are but loosely attached, and may, both in health and disease, be considerably removed from their natural position, we perceive how cautious we ought to be in affirming, because a tumor exists in a certain region, that it is always, or necessarily, connected with the viscus which that region naturally contains. When the external characters of such tumors are obscure and inconclusive, we are told that the function of the affected organ will be always so much deranged as to enable us to establish a satisfactory diagnosis. This is by no means the case,—tumors having been often met with in the epigastric and umbilical regions, unaccompanied by any clear or satisfactory symptoms, but which were found, on dissection, to depend on organic diseases of the stomach. Were we, therefore, to recognize a large globular tumor below the umbilicus, we might be apt to conclude that, from whatever part it originated, its low position, and the absence of symptoms indicating an affection of the stomach, distinctly showed that this viscus was not involved. But again,—we know that the stomach often descends considerably into the abdomen; and that, on this account, tumors attached to it have, from their depending position, been mistaken, not unfrequently, by experienced practitioners, for other diseases,—a case of which is mentioned by Dr. Monro. There are also several interesting cases of large tumors of the stomach occupying different regions of the abdomen, detailed by Dr. Seymour, in the fourteenth volume of the Medical and Surgical Transactions of London. In some of these, the absence of well-marked symptoms, and the unusual size and situation of the tumors, did not lead to a suspicion, that the stomach was implicated, until this was ascertained by dissection. The pyloric orifice was the part affected, and the tumors presented the malignant characters of the encephaloid disease.

The difficulty is still farther increased, because, in all the artificial divisions of the abdomen, there is situated, not a single organ, but a variety of parts—in any one of which the tumor may be situated. If it exists in the centre of the abdomen, it may arise from the peritoneum, the omentum, the intestines, the mesentery, the stomach, &c.; if in the hypogastrium, from sources not less numerous and obscure. There is not, in fact, a more difficult and uncertain part of medical practice, than to distinguish between the different tumors daily to be met with in the abdomen, or to obtain any thing like conclusive or satisfactory evidence as to their origin and connexions. It is this uncertainty of diagnosis, so generally felt and acknowledged, that renders the question regarding the propriety of surgical interference so interesting and important."

There are few in extensive practice—none who have spent much time in hospitals, to whose recollection illustrations of the difficulties, pointed out by Dr. Macfarlane, will not occur. Dr. Macfarlane's chief object, in relating the cases we shall now detail, is to shew the dangers attendant on the operation of gastrotomy. But this question is, to our minds, of less importance than the object of pointing out to practitioners, the various modes in which their diagnosis may be rendered fallacious. Cases are of great value in diminishing difficulties of this description, for the mind, being aware of errors that have occurred, is on its guard against their commission again.

Our author divides his subject thus: He considers—

1st, Tumors confined to the abdominal parietes.

2nd, Tumors depending on disease of the peritoneum, omentum, or mesentery.

3d, Tumors arising from alvine concretions; and,

4th, Ovarian Tumors.

### I. Tumors confined to the Abdominal Parietes.

Such tumors are not very rare, yet no good account of them exists, at least we have not seen any. We have witnessed, however, some signal blunders made as to their nature.

Case. 1—*Cystic Sarcoma of the Abdominal Parietes.*

H. M. æt. 4, admitted Oct. 11th, 1831. There was situated in the right inguinal region, and extending downwards in the direction of Poupart's ligament, a prominent ovoid tumor, about four inches in diameter. It felt firm towards the margin, which was ill-defined, but was tuberose on the surface, and in several places so elastic as to resemble a collection of cysts containing fluid. It was freely moveable over the subjacent parts, but was firmly and intimately fixed to the integuments, which at one point had a blueish colour. When first observed, at birth, it was about the size of a field-bean, but did not increase much till about nine months before he was brought to the Infirmary, after which it became the occasional seat of pain.

The tumor appeared to be partly solid, partly fluid, and was evidently seated in the abdominal parietes. Its extirpation was, therefore, resolved on, and soon afterwards performed. The wound healed after a partial suppuration, and the boy was dismissed on the 14th of November.

The tumor was found to consist of an assemblage of cysts varying in size from a filbert to a pea. Several of the most superficial communicated with each other, and presented a honey-comb appearance; but, more deeply seated, each cyst was entire, and apparently separated from its neighbour by cellular tissue. They were covered externally by a fibrous, and lined internally by a smooth, serous membrane, and filled with a dark-coloured fluid.

"This variety of tumor, (says Dr. Macfarlane) is met with more frequently in some parts of the body than in others; and in advanced life it occurs more frequently than in youth. It is unaccompanied by pain, and the integuments placed over it retain their natural colour until the fluid is close to the surface, when a slight blue tinge is observed. It is, in general, easily removeable by operation; and there is almost no risk of its reproduction. When there is only one cyst, and that one is small in size, the disease may be cured by evacuating the fluid, and producing inflammation and cohesion of the secreting surfaces; but, when the tumor is large, or is composed of several cysts, the complete removal of the diseased mass by the knife is always necessary. I have seen severe local inflammation and high constitutional excitement produced by puncturing such tumors, with a view to their cure by inflammation or suppuration; and I would deprecate this practice, as less certain in its effects, and more hazardous to the patient, than that of simple extirpation.

With the exception of the ovaries, there would seem to be a greater tendency to the formation of encysted tumors in the cellular texture than in any of the other tissues of the body. Were the cells of this texture to become obliterated by adhesion or otherwise, the natural serous secretion may probably be so gradually augmented as to form a distinctly encysted tumor; at least, I have seen more than once appearances which rendered this supposition by no means improbable. The same explanation may also hold good in many of those cases in which there exists a congeries of cysts, distinct from or communicating with each other."

Dr. Macfarlane makes a remark upon chronic abscesses of the abdominal parietes, to which we would direct attention.

Chronic abscesses, he observes, sometimes form between the layers of the abdominal muscles, or immediately exterior to the peritoneum. When these are large and ill-defined, it is hardly possible to determine, until the matter approaches the surface, whether the tumor is situated in the parietes, or arises from some of the deeper-seated parts within the cavity of the abdomen.

We have witnessed eight or ten instances of chronic abscess, situated in some part of the abdominal parietes. The majority of those cases were misunderstood prior to a late period, and advanced stage of the complaint. A discussion on this subject took place lately at the Westminster Medical Society. The junior Editor of this Journal related to that body the particulars of several cases he had seen. In one, the seat of the collection of matter was found, on dissection, to be the cellular tissue between the peritoneum and the transversalis muscle; in another, it was between the internal oblique and transversalis, whilst, in the rest, either an examination after death was not obtained, or, if obtained, was unsatisfactory.

We have seen these abscesses point in the loins, at the side of the abdomen, in the groin, and over the outer abdominal ring. Some of the patients thus affected have died, others, perhaps the majority, have recovered. The last example of this not very unfrequent affection that occurred to us was in the person of a gentleman, of some eminence in the musical world. The abscess, in this instance, was seated in the left iliac region. A physician of some eminence had been in attendance, and had no conception that an abscess of the abdominal parietes existed. An opening, however, shewed that this was the fact. The patient was extremely ill, but he rallied, and was sent into the country for the benefit of change of air. We are unacquainted with the result, if, indeed, a termination has arrived.

The next kind of tumor of the abdominal parietes to which Dr. Macfarlane draws attention, is one of an encysted kind.

CASE 2.—*Encysted Tumor of the Abdominal Parietes—Puncture—Peritonitis—Death.*

A woman, æt. 40, had the middle and inferior regions of the abdomen occupied by a smooth, firm, globular tumor, of great size. It had existed for two years before she applied to a surgeon ; but she could not point out the situation in which she first observed it, nor give any satisfactory account of its progress. An obscure feeling of fluctuation existed in the centre, which projected in a somewhat conical form ; and the integuments were so tense, and the boundaries of the tumor so ill-defined, as to render it impossible to obtain a knowledge of its connexions. It was the frequent seat of acute pain ; and, as it increased in size, it appeared by its bulk and pressure, to impede the action of the bowels, and give rise to frequent attacks of colic. Very contrary opinions of the nature of the tumor were entertained; but it was punctured with a trocar, and several pints of fluid, of the colour and consistence of mucilage, were evacuated. Peritonitis supervened, and proved fatal in five days. On dissection, the fluid was found to have been contained in a large cyst, formed internally by the peritoneum, and externally by the abdominal muscles.

Dr. Macfarlane is of opinion, that an earlier puncture would have been attended with less danger. This is probably correct. He alludes to, and condemns, the practice of employing a stimulating injection, recommended by M. Lisfranc. The practice is too hazardous to be followed, without mature reflexion, by any prudent surgeon.

Abscesses, as Dr. Macfarlane observes, frequently form in the iliac regions, enlarge slowly to a great size, and, in the female, are occasionally mistaken for ovarian tumors. The diagnosis is often obscure. M. Dupuytren, as our readers will remember, has published a considerable collection of cases of abscess in the right iliac region ; in this, it is not very unfrequently connected with the lodgement of a foreign substance in the cæcum. The following is a characteristic case.

CASE 3.—*Chronic Abscess in the Right Iliac Fossa, bursting externally—Artificial Anus—Death.*

Mrs. A. æt. thirty-eight, complained for several months of a dull pain in the right iliac region, before any swelling or constitutional derangement was perceptible. A tumor was by-and-bye discovered, which appeared deeply seated, was smooth and firm to the feel, and without fluctuation. It continued for six months without undergoing any perceptible change; but her strength began to decline, and the countenance to assume a sunk and haggard appearance. She had now frequent attacks of pain in the belly. The pulse was accelerated, the bowels were irregular, the stomach irritable, and, as the tumor advanced, the right leg became œdematous. Soon after the commencement of these symptoms, fluctuation was indistinctly recognized in one part of the tumor, which now filled the iliac fossa, extended considerably above the crest of the ilium, as also backwards to the spine, and projected about two inches beyond the level of the surrounding parts.

Dr. Macfarlane was uncertain whether the tumor was occasioned by an encysted disease of the ovary, or a chronic abscess. He proposed a cautious puncture. The patient refused to submit to this—pointing, and ulceration of the integuments ensued—an immense discharge of pus took place—in three days afterwards, fæces and flatus were discharged through the opening—and in eight days the patient died.

It was found, on dissection, that a large thick cyst occupied the right iliac and lumbar regions, and extended into the pelvis : it also surrounded the cæcum, in which there was an ulcerated opening, capable of admitting a finger.

"Women, in the puerperal state, are liable to the formation of abscesses immediately above the groin. These, when slow in their progress, and confined to the cellular texture of the iliac fossa, are difficult to be discovered; but, when they rise above Poupart's ligament, and separate the peritoneum from its connexion with the parietes of the abdomen, they are more easily recognized. We also meet with abscesses in the same situation from inflammation and suppuration of the ovarium.

Dupuytren recommends that all these deep-seated abscesses should be left to nature, and allowed to burst, as they generally do, into the bowels, vagina, or bladder. I think, however, that as soon as the symptoms are well-marked, much mischief may be prevented by puncturing them, and evacuating the matter,—a practice which I have frequently had recourse to with decided advantage."

We perfectly agree with Dr. Macfarlane in the last piece of advice. A moderately early opening of these serious chronic abscesses is generally advisable.

Case 4. *Organized Sarcomatous Tumor between the Layers of the Abdominal Muscles.—Extirpation.—Cure.*

W. T., æt. twenty-two, 25th September, 1831. There was situated in the right iliac region, nearer to the spinal column than the umbilicus, a smooth ovoid tumor, about the size of the fist, which had a hard cartilaginous feel, admitted of hardly any motion, and was apparently attached to the floating end of the twelfth rib. It projected nearly an inch beyond the level of the abdominal integuments, and was distant six-and-a-half inches from the umbilicus, four inches from the spinous processes of the vertebræ, and four from the anterior superior spinous process of the ilium. The fingers could be partially insinuated under its anterior margin, which was slightly irregular, and from which firm bands were felt passing in various directions.

When the tumor was first observed, two years ago, its origin being attributed to severe exertion, it was about the size of a field bean. It gradually increased, and became so painful as often to prevent sleep,—the pain being not only situated in the tumor, but also extending across the abdomen, and along the right thigh.

The majority of Dr. Macfarlane's colleagues thought in consultation that the tumor was too deeply attached to admit of a safe and successful operation. Mercurial inunction, &c. were employed without avail, and at a second consultation, the removal of the tumor by the knife was agreed to.

The tumor was extirpated with great ease. On making an elliptical incision, eight inches in length, and dissecting off the investing integuments, the tumor was still covered by a layer of muscular substance, which was found to be the external oblique. When this was divided, the tumor came into view, and was found resting on, but not attached to, the ribs. It was easily separated from its posterior attachments by the finger, and made to start from its deep position by pressure. It was about the size of a lemon; had a greyish colour, not unlike half-bleached wax; was in some parts semi-transparent, and exhibited a smooth, compact texture,—its centre being fibrous. It was covered by a firm membrane; and was found, when analyzed, to be chiefly composed of albumen.

The wound healed well, and the patient was dismissed cured on the 15th of November. Dr. Macfarlane appends some judicious observations on the diagnostic marks of the tumor, or rather of its seat and its attachments. But these may be omitted.

Case 5.—*Fibro-cartilaginous Tumor between the Layers of the Abdominal Muscles—extirpated with part of the two Inferior Ribs—Death.*

Mrs. R., æt. twenty-six, admitted on the 18th January, 1832. In the right iliac region of the abdomen there was situated a tumor about the size of the fist. Its posterior margin was distant from the spinous processes of the vertebræ two inches, and it passed obliquely downwards and forwards to near the anterior superior spine of the ilium. It appeared to be attached to the two inferior ribs, and for the space of two inches to the crest of the ilium. It had a hard cartilaginous feel, was of an oblong shape, and measured six inches in the greater and five in the lesser diameter. It projected from the side of the abdomen somewhat prominently, was covered by healthy integuments, admitted of limited motion, and at the lower edge the fingers could be passed under it. It occasioned severe pain, especially during the night, which extended to the thigh and prevented sleep. Its origin was attributed to a fall against the edge of a table three years ago. When first observed, it was as large as a pigeon's egg, and was situated over and apparently fixed to the ribs. Her health had varied considerably, and she had employed a variety of local and constitutional remedies without the slightest benefit.

The patient had been in the hospital a year previously, when the tumor was smaller, more moveable, and less painful. Subsequently it became more fixed. The patient was anxious for its removal, and Dr. Macfarlane was convinced that it was placed between the layers of the abdominal muscles, and that it might be removed without laying open the cavity of the abdomen, or wounding the peritoneum. At the same time he was aware of and pointed out to the patient the risk of peritoneal inflammation. She consulted many gentlemen, obtained, as usual, various opinions, and it was not until the 19th January, 1835, and when the tumor had acquired the characters already fully mentioned, that she made up her mind to submit to the operation.

" She was laid on her left side, under which a folded pillow was placed. An incision was made over the centre of the tumour, commencing an inch from the spinal column, and extending obliquely downwards and forwards for about two inches beyond the anterior superior spine of the ilium. The integuments were dissected back, and the external oblique muscle exposed. In order to ascertain how many muscles covered the tumour, I cut freely down over its lower margin, and found it to be situated betwixt the internal oblique and transversalis. It adhered so intimately to both these muscles, that its separation from them was impracticable. I therefore divided the transversalis, with great caution, at the lower part of the wound. The finger was then passed between it and the peritoneum, and the internal oblique and transversalis divided close to the lower and outer edge of the tumour, which had to be separated also from the iliacus internus, and from the psoas muscle, under the edge of which it dipt. Its attachment to the floating ends of the two inferior ribs was so intimate as to render necessary the removal of these parts along with the tumour. The finger was passed under the ribs to separate the subjacent soft parts from them, which was done with ease: they were then divided close to the tumour with Liston's bone forceps. The operation was finished by detaching the anterior part of the tumour, and separating the peritoneum from its base. Three arteries were tied; the largest of which was the epigastric. Not more than six ounces of blood were lost. The cavity that remained could contain the fist. Its boundaries were distinctly seen, and the peritoneum found to be uninjured. Four sutures were introduced, and the wound dressed with straps and compress, over which a broad bandage was applied."

On examination, the tumor, which was of an oval shape, and somewhat flattened, had a smooth surface, but felt exceedingly hard. It was of a fibro-cartilaginous structure, and was with difficulty divided with a scalpel. The extremities of the two ribs had penetrated fully an inch into its substance, and were firmly attached to it. It was evident that the tumor had not originated from the ribs, but had only become attached to them during its growth.

The succeeding diurnal reports we need not dwell on, our object being rather to point out the characters and symptoms of abdominal tumors than to notice the common sequelæ of operations. The patient became affected with the usual symptoms of peritonitis, and died in the night of the 22d, eighty-one hours after the removal of the tumor.

On inspection, the common marks of peritonitis were discovered, the peritoneum, however, having been uninjured in the operation. A small quantity of blood was extravasated into the cellular tissue between the peritoneum and the iliac muscles.

The two preceding cases are both interesting. They shew the occasional occurrence of a morbid growth, of a fibro-cartilaginous character, between the layers of the abdominal muscles, and they shew too the necessity of forming a correct diagnosis early, in order that an operation may be practised before the tumor has acquired attachments that render its removal difficult and dangerous.

### II. Tumors of the Peritoneum, Omentum, and Mesentery.

Tumors, Dr. Macfarlane observes, are not unfrequently developed in the peritoneum, grow to an immense size, and produce urgent symptoms; yet the most accurate examination will seldom enable us to ascertain their seat or attachments. Sometimes these are confined to a small portion of the peritoneum, covering the abdominal muscles: they are, however, oftener found in connexion with extensive disease of the omentum.

Case 5. *Disease of the Omentum simulating Enlargement of the Ovarium.*

Dr. Macfarlane was requested to visit Mrs. O., æt. 49, on account of ascites, complicated with a tumor in the abdomen. It had commenced four years previously as a small moveable tumor in the right side of the belly, below the umbilicus, which did not give her pain nor increase in size for three years. She had then an attack of peritonitis, from which she recovered with difficulty, and was afterwards subject to acute pains in the abdomen,—nausea, vomiting and constipation. The tumor now increased rapidly; fluctuation was distinctly perceptible; she became emaciated; the pulse quick and feeble; the tongue red and parched; the urine scanty; and she had cough and dyspnœa.

Dr. Macfarlane drew off the water in the abdomen, and was then enabled to institute a more precise examination of the tumor. It was about the size of a child's head, and placed in the anterior and inferior part of the abdomen. It had a nodulated feel; could be moved from side to side; and did not appear to be attached to any of the subjacent viscera. The fingers could be pushed under it; and when it was raised up as far as the relaxed state of the abdominal integuments would permit, it was found fixed to the pelvis, by a pedicle about the thickness of the wrist. From its upper edge to about the arch of the colon, several other tumors of different sizes were felt; and, in connexion with these, there was a hard ridge, about the thickness of the finger, passing obliquely across the abdomen.

"At first, I was inclined to believe that the right ovarium was the seat of the disease, and that the fluid had accumulated, not in the cavity of the peritoneum, but in an ovarian cyst, of which the nodulated tumours formed a part. On examining more minutely, however, I ascertained that some of the small tumors were attached to the parietes of the abdomen. The integuments were so relaxed and attenuated by long-continued distention, that I was able to pull them out to a considerable distance from the subjacent viscera; and, by grasping them between the extended hands, and rubbing the one against the other, the irregularities alluded to were distinctly recognized. I was now satisfied that the peritoneum lining the abdominal muscles was affected, and that its surface was covered with tubercles, forming the disease so well described by Dr. Baron;* but I still considered the large tumour connected with the pelvis to be an enlarged ovary."

The water re-accumulated in the abdomen, and in about two months the patient died.

*Dissection.*—In the cavity of the abdomen there were about ten pints of a dark-coloured viscid fluid. The peri-

* "See Enquiry, illustrating the nature of Tuberculated Accretions of Serous Membranes, &c.—London, 1819."

toneum covering the abdominal muscles was much thickened, of a dirty yellow colour, and studded over with tubercles of different sizes. The intestines were hid by a large mass of tumors, extending from the epigastrium to the pelvis, arising, not from the ovaria, which were healthy, but from a diseased state of the omentum. At the upper part, the mass retained the natural attachments of the omentum to the stomach and colon; but inferiorly, it was fixed deep in the pelvis by preternatural adhesions, forming the thick stalk which was mistaken, during life, for an ovarian pedicle. The omentum was hard, and, in some places nearly cartilaginous; and the whole mass weighed nine pounds.

Dr. Macfarlane points out the liability of such a morbid growth to be mistaken for ovarian tumor, and the serious consequences that would follow from gastrotomy performed for that disease. The taste for gastrotomy is *now* gone by, and little need be said upon that head. Dr. Macfarlane quotes from Andral a case somewhat similar to the preceding. In that, after the existence of obscure symptoms of abdominal and uterine irritation for several months, a round tumor was discovered, rising from under the pubes and reaching to the umbilicus, which was supposed to be the uterus. It gradually enlarged, laterally, so as to occupy both iliac regions,—became irregular on the surface, and acutely painful on pressure. On dissection, the tumor was found attached above to the stomach and colon, and below to the uterus and its broad ligaments. It consisted of a scirrho-cartilaginous enlargement of the omentum.

"This disease of the omentum, when not accompanied by ascites, has also been mistaken for extra-uterine pregnancy. I have met with two cases of this kind,—one of which I shall detail very shortly; the other I had only an opportunity of examining once. This patient was seen by an immense number of medical men; and, when in Edinburgh, she was requested to submit to an operation, which she declined. I learned afterwards that the nature of the disease was correctly ascertained by dissection."

CASE 7.—*Disease of the Omentum mistaken for Extra-uterine Pregnancy.* A woman, æt. 41, had menstruated twice after weaning her fourth child,—when this secretion ceased, and did not again return. She then began to complain of dull pains, with a feeling of weight in the belly,—of nausea, occasional vomiting, flatulence, and constipation. The abdomen became gradually more and more prominent, the mammæ enlarged, and she felt confident that she was pregnant. At the end of the sixth month from the commencement of these symptoms, the swelling became stationary; and in three weeks it had declined considerably. She had now frequent and severe attacks of pain in the abdomen, with increased disturbance of the bowels. Emaciation took place rapidly; the countenance became sharp and anxious; the pulse quick and feeble; the tongue dry and florid; and the abdomen was still more prominent than natural, particularly in the hypogastric, umbilical, and left hypochondriac regions.

On examination, an ill-defined tumor, of a globular form, and about the size of an orange, was discovered, a little above and to the left of the umbilicus; whilst immediately under, and apparently in connexion with this, three or four ridges, separated from each other about a quarter-of-an-inch, were felt, and could be traced obliquely across the abdomen for nearly three inches. These appearances, when viewed in connexion with the history of the case, led to the belief that extra-uterine pregnancy existed. An operation was proposed and even agreed to, but Dr. Macfarlane having seen the same appearances in a case of diseased omentum, dissuaded the patient from submitting to the knife.

In about three weeks she died. On dissection, the omentum was found so diseased as not to retain the slightest vestige of its original structure. It consisted of globular tumors of various sizes,—the largest of which was situated in the left hypochondrium, and had been mistaken, during life, for the head of a child. Their texture was fibro-cartilaginous, as were also the ridges, which extended along the whole breadth of the diseased mass, and which resembled the ribs of a child when examined through the parietes of the abdomen. The omentum was also attached to the intestine by old adhesions.

"Gastrotomy may be performed with success when the fœtus has passed through a rupture of the uterus into the cavity of the abdomen. When this takes place, the symptoms which it excites are seldom very equivocal. It appears during labour, and is generally preceded by violent uterine action,—which is immediately followed by a complete cessation of the parturient efforts, and alarming collapse. The absence of the presenting part of the child, on examining per vaginam, and the irregular flattened feel of the abdomen, in which the outlines of the fœtus can be traced, will also materially guide the diagnosis. When the hand can be introduced, however, through the lacerated opening into the abdomen, and the child extracted by the vagina, no surgeon would think of having recourse to gastrotomy.

This operation, although strongly recommended, is, I think, of more questionable utility when the fœtus has been extra-uterine from its commencement, and has grown either in the fallopian tubes, in the ovaries, or in the cavity of the abdomen. We can rarely obtain symptoms sufficiently characteristic of this form of extra-uterine fœtation: but, although an operation were performed, and our opinions found correct, we have difficulties still to encounter in opening the pouch, extracting the child and placenta, and arresting the hemorrhage, which will be more apt to lead to a fatal result, than were the case left to nature. The fœtus may remain for a great length of time in the abdomen without exciting very urgent symptoms; or it may contract adhesions to the walls of the abdomen, to the bowels or bladder, and be discharged by ulceration."

### III. Tumors from Alvine Concretions.

Alvine concretions occasionally give rise to tumors, which present the characters and occasion the effects common to many or almost all abdominal growths. Their diagnosis is frequently obscure. The following are curious instances of the facility with which errors may be committed.

"I recollect having had under my care, when a student of medicine, one of the city paupers, who had a tumour in the right side of the abdomen, about the size of a fist. From the account she gave of it, its apparent connexions, and the accompanying symptoms, it was supposed to be a concretion in the colon. She stated that, when it was first discovered above the right ilium, she could move it upwards and forwards, in the direction of the colon, to about the middle of its arch, but, as it increased in size, it became fixed between the crest of the ilium and the false ribs. She had severe pain and troublesome constipation, followed by repeated attacks of peritonitis.

After several consultations, it was agreed to lay open the abdomen and colon, and remove the concretion. I was present at the operation, which was performed by a respectable surgeon, with the concurrence of the first medical authorities of the place. When the abdominal integuments were divided, a large cyst came into view, which was opened, and found to contain hydatids. It was extensively attached to the concave surface of the liver, and, of course, could not be extirpated. It had so pressed on the ascending colon as to produce an impediment to the passage of the feces, and attacks of peritonitis, in every respect as well marked as if the tumour had existed within the cavity of the bowel.

Tumours of various and dissimilar kinds have been mistaken for alvine concretions. In one case detailed in the Edinburgh Medical and Surgical Journal, (No. 33, p. 112,) an operation was strongly advised, but fortunately not performed, as the disease turned out to be scirrhus of the pylorus."

A biliary calculus, lodging in the bowels, may become the nucleus of a concretion.

Case 8.—*Alvine concretion in the Ileum—Peritonitis—death.*

A ploughman, æt. 38, had an attack of jaundice, with acute pain in the epigastric and right hypochondriac regions, which was removed by emetics, purgatives, and venesection. He continued in good health for three years, when he had occasional colic pains and constipation, for which purgatives and enemata were employed with advantage. These symptoms increased, and gastro-enteritic fever supervened. Immediately under the umbilicus, where he had a constant feeling of weight and distention, a solid but ill-defined tumor was discovered, which was slightly moveable from side to side. He had a violent attack of peritonitis, the flatulent swelling increased so as to conceal the tumor, and the patient shortly died exhausted.

On inspection there were found the marks of peritonitis. The small intestines increased gradually in size and thickness from the stomach downwards. The ileon was excessively distended, and contained, near its termination, a globular tumor, as large as an orange. This was found, on slitting up the bowel, to be an alvine concretion, of a dark brown colour, rather rough, and porous externally, but, internally, of a more dense and compact texture,—the nucleus, consisting of a yellow-coloured biliary calculus, about the size of a kidney bean. The mucous tissue of the ileon was extensively ulcerated, and the calibre of the gut contracted immediately under the site of the tumor.

In this case the symptoms were tolerably well marked, yet no satisfactory diagnosis could be formed. Of the advice to cut down upon the colon when alvine concretions are suspected we need say nothing, The taste for vivisections has declined within this year or two.

### IV. Ovarian Tumors.

As Dr. Macfarlane properly remarks, the disease of the ovaria in early life, is usually the formation of cysts; because at that period the organ contains a number of vesicles. In later age the ovary shrivels, and then more solid enlargements prevail. Sometimes, he continues, the first appearance of disease in the ovary consists in the formation of a cyst, which gradually enlarges, so as nearly to fill the abdomen; but more frequently, sarcomatous enlargement takes place to some extent before any serous or gelatinous fluid is secreted. When this occurs, the tumor, at an early period is smooth, compact, and globular; but, as it enlarges, it becomes tuberose, from the effusion of fluid, and the consequent development of cysts within the ovarian substance.

In the first case related by our author the left ovarium was occupied throughout by a solid enlargement. We see nothing in it worth recording.

The second case is more interesting, being a specimen of malignant alteration of both ovaries.

Case 9.—*Malignant Enlargement of both Ovaria.*

A. M. æt. forty-three. The abdomen, which was as large as is usually observed in the ninth month of utero-gestation, was completely filled with a smooth and apparently solid tumor. By relaxing the abdominal muscles, which were unusually thin, and by placing the patient in a favourable position, Dr. M. was able to push his hands under the tumor, raise it slightly from the subjacent viscera, move it a little from side to side, and make the abdominal parietes glide over it. This appeared to show that no adhesions existed between it and the viscera or parietes of the abdomen; but it was impossible to ascertain the nature or extent of its attachments to the pelvis. It filled this cavity, and of course admitted of very little motion. The disease had commenced nine years previously as a small moveable tumor above the right groin. Two months previously the disease had become much aggravated in consequence of her receiving a blow upon the part. In a month after the date of the report she died.

" *Dissection.* The tumor was slightly attached to the parietes of the abdomen and to the omentum; but it adhered very intimately to, and was with difficulty separated from, both sides of the pelvis, the fundus of the bladder and uterus, and the upper part of the sacrum. It was found to arise from the right ovarium, and had for its pedicle the broad ligament and fallopian tube, which were greatly enlarged, hard and tuberculated. The fundus uteri was also irregularly enlarged and possessed the stony hardness and fibrous appearance of carcinoma. The tumor was of a solid texture, except towards its posterior and inferior part, where it contained several cysts, filled with a dark-brown fetid fluid, in which were small coagula of blood. The lining membrane of these cysts, which were of different sizes, was covered with soft, purple-coloured, spongy excrescences; and the solid portions of the tumor surrounding them were softened and mixed with fetid matter. The left ovarium was likewise enlarged, and adhered to the corresponding side of the pelvis and to the large tumour.

In this case, the ovaria, right fallopian tube, and fundus uteri, were involved in the same disease, which was probably malignant.

As Dr. Macfarlane correctly observes, the presence or absence of burning pain is no criterion of the simple or malignant nature of the tumor, the pain being dependent on the existence of slight peritoneal inflammation.

Dr. Macfarlane has witnessed during the last fifteen years above forty cases of ovarian tumors, and he possesses the notes of fourteen dissections performed by himself. The following particulars are interesting in connexion with the question of extirpation.

"When the disease was first noticed, two of the fourteen patients were under thirty years of age; four were from thirty to forty; five from forty to fifty; and three above fifty. Before the disease proved fatal, it existed in two cases four years; in three, eight years; in one, nine; in four, eleven; and in other four, from thirteen to eighteen years. In four cases, the tumour was confined to the right ovary; in seven, to the left; and in three, both were implicated. In one, the ovarium was distended into a large cyst, without any appearance of solid growth, and was, of course, mistaken during life for ascites; in nine, chronic enlargement existed, in combination with one or more cysts containing fluid, (six of these were tuberose, and three smooth on the surface); and the remaining four were solid throughout. In twelve of these cases, adhesions, more or less intimate and extensive, existed between the tumours and the parietes and viscera of the pelvis; three of which had likewise become adherent to the abdominal parietes, omentum and intestines. Only two were free from preternatural adhesions; and in these the tumours were attached to the broad ligament by a slender pedicle. In three of the twelve cases, it appeared that the adhesions might have been divided without much risk or difficulty; but in the remaining nine, this procedure was altogether impracticable, and could not have been accomplished without imminent danger to all, and certain death to some. In eight of the adherent cases, the basis of the tumour was thick and broad,—the size of this part appearing to depend more frequently on the extent and intimacy of the secondary attachments to the pelvis, than on the magnitude of the tumours.

Before stating the objections which, from these and other considerations, I have been led to form against the operation of gastrotomy for the removal of ovarian tumours, I shall allude very briefly to the results of some of the published cases in which this treatment was employed. These remarks shall be confined to twelve cases, three of which occurred to Dr. M'Dowal, of Kentucky, in America; four to Mr. Lizars, of Edinburgh; and five in Germany, to Deiffenbach, of Berlin, Hopfer, Chrysmer, and Martini. Of these, four died and eight recovered. In nine, the tumours adhered, more or less extensively, to the contiguous parts: in four of these, the adhesions were divided, and the tumours wholly removed; in one, only a part of the disease was extirpated; and in the remaining four, the extent and intimacy of the existing adhesions prevented the operations from being completed. In one of the cases, the abdomen was opened, and no tumour found: this patient fortunately recovered. Besides the extent of the adhesions, the operator was deterred from removing the tumour in another case, by the number and size of the arteries, which were seen to pulsate violently in the pedicle."

Gastrotomy is now so little likely to be hastily performed that we need not add the doctor's arguments against it. We beg the attention of our readers to the preceding cases, containing as they do much valuable practical information.

---

### FRENCH OPINIONS UPON ENGLISH HOSPITAL SURGEONS AND SURGERY.

The constitutional vanity of a Frenchman has long been a matter of admiration and amusement to surrounding nations. There is no country like La belle France—no people like the brave Français—and every thing he sees or hears, or reads of, satisfies the Frenchman more and more of his incomparable superiority over the remainder of mankind. Madame de Staël hit off the character of the Gallic traveller to the life. Her Comte d'Erfeuil is a perfect representative of the genus.

"Il parcouroit chaque ville, le guide des voyageurs à la main; il avoit à la fois le double plaisir de perdre son temps à tout voir, et d'assurer qu'il n'avoit rien vu qui pût être admiré, quand on connoissoit la France."

Some one, we forget who it was, remarked that the French are a very ignorant people, for they know scarcely any thing of what other nations do. The observation is a very just one; for, although some brilliant exceptions may be found, the sçavans of France are so perfectly contented with their monopoly of all knowledge, that they look with sovereign contempt at the tiny folks, who, in England, and Germany, and Italy, are so credulous as to imagine that they are not entirely destitute of some small portion of learning and science.

A M. Baumés, a Comte d'Erfeuil in his own small way, has lately, it would seem, paid a visit to the English hospitals. On his return to France, he could do nothing less than expose the utter darkness of the English surgeons, and the raw, uncivilized state of English surgery. Accordingly, he wrote a series of letters in the Gazette Médicale, exposing, in a candid and an able manner, the imbecility of M. Jean Bull.

These letters have been noticed in two of our contemporaries—the Medical Gazette, and Dr. Ryan's Medical and Surgical Journal. The former is inclined to be merry with M. Baumés; but the writer in the latter is delighted at the exposé of his countrymen.

M. Baumés, it seems, is a citizen of Lyons, where he is chirurgien-en-chef of a hospital. Now think of a chirurgien-en-chef of Lyons, condescending to visit so inconsiderable a town as London. Dr. Ryan's journalist very properly dwells upon the gratifying circumstance, and ingeniously remarks, that a man of such importance is not likely "to cast any sheep's eyes" at the hospitals of our city. This reasonable supposition has probably been fulfilled, as we have not yet heard of any such eyes having been discharged at Mr. Lawrence or Sir Benjamin Brodie, while peripateticising in St. George's or Bartholomew's. But, though M. Baumés has thrown no sheep's eyes, he has flung much dirt, and our contemporary, like those industrious little boys that the curious traveller may see at horses' tails, trailing a small barrow, and carefully collecting their valuable dung—our contemporary, we say, has put in his small barrow M. Baumés' mud, and strange-looking mud it is.

In the first place, M. Baumés is shocked to observe that, since the visit of M. Roux—a fifth part of a century, the art of surgery has undergone scarcely any change in England. What an excellent thing is a microscopic eye. We, in our simplicity, thought we had observed some important changes, even within these last ten years—we thought that a great deal had been done to increase our knowledge of the different varieties of tumors—to correct our notions and regulate our practice with respect to hernia—to distinguish the diseases of the breast and testicle—to define our acquaintance with the different fractures and dislocations—and we dreamed that much, very much, had been done, in the advancement of our acquaintance with the diseases of the urinary organs and the joints. In all these departments of surgical science, we were actually so very ignorant and weak, that we imagined our brothers of France were behind us. Point du tout. They are in the van—they are out of sight, so far out of sight, that we cannot even see the dust that their march raises. M. Baumés is émerveillé at our halt—he is "struck all of a heap" at the sorry figure cut by our miserable hospital surgeons. The diseases of the joints are not at all understood by Sir Benjamin Brodie—his pathological investigations are mere moonshine—and the only real acquaintance with these maladies is to be found in France. Hear M. Baumés :

"He has seen different kinds of articular tumors, white swelling, for instance, increase and aggravated with rapidity, ultimately rendering an operation necessary, when a rational treatment might have superseded it;" and "any who have observed their (the English hospital surgeons') practice for a given time may readily remark, that amputation is frequently had recourse to in cases that, in France and other countries, would assuredly be cured by following the more rational means of pathological physiology."

How delightful it is to see a new light poured in upon a subject—to observe how it dispels old and prevalent errors, as a candle routs a host of bugs. It was generally believed, until M. Baumés asserted the contrary, that the French were the people for unnecessary operations—the French hospitals the spots where limbs were sacrificed, that in this country are saved. Such was the remark of every English surgeon and physician, nay, of every English student of medicine, who witnessed the practice of the Continental hospitals. But this was all a pure mistake—our students and our surgeons were blind or jealous—they were thinking of what they had seen in England—not of what they then witnessed abroad. Oh yes! it was reserved for M. Baumés, and the writer in Dr. Ryan's Journal, to discover the delusion ; and they have discovered it. Par nobile fratrum!

"Again," says the English echo of the Gallic cock, "to shew how 'the operation' is always uppermost in the mind of your conceited hospital surgeon, how much rather he makes a case an opportunity for the display of his operative powers, than of his conservative capabilities, it is only necessary to say that when M. Baumés observed the ill-placed treatments of these articular inflammations to several of those who treated them, the answer he got was, that 'if they put off the operation too long a time, that is, if strong febrile symptoms supervened, the operation did not succeed;' whereas the very fever they feared themselves, originated with their calomel, their iron, antimony, and similar irritants ordered at a risk during one of their flying visits. 'I have seen (says the writer in another place), in several hospitals, patients affected with articular tumors, with caries of the bone, fistula, abscess, &c. to whom amputation was proposed; and in the meantime, under pretext that a great degree of debility existed, wine, tonics, and various cordials were freely administered, although it was at the same time evident that there was extreme irritation of the gastric organs, in great part consequent on the evacuant, aperient, mercurial and other treatment that had been employed for the purpose of getting rid of a complaint which they had thereby increased.'"

So those ignorant fellows, our hospital surgeons, first of all treat an articular inflammation by calomel, iron, antimony, and *similar* irritants. Really we can see little likeness in the operations of iron and of antimony; and we do not think the latter, at all events, very likely to produce a fever. But pardon, M. Baumés, antimony will produce a fever—a real fever—a gastro-enterite. Aye, there's the rub. Our Brodies and Coopers are not of the new light—they are not of the élite of the école physiologique—they are not Broussaisans—they don't order leeches to the epigastrium, for scrofulous diseases of the bones of the knee. Stupid and wicked folly! Well may the writer in Dr. Ryan's journal elegantly observe :—

"Here there are some nuts for our pure surgeons to crack; the writer does not get his information second-hand: what he relates he has witnessed 'in the practice of the first surgeons in London'—*first* surgeons!!"

First surgeons indeed—no, this well-informed reviewer and M. Baumés are the real chirurgiens-en-chef. See how they would manage a white-swelling—see how they would apply the great physiological theory. Unfortunate patients, to be deprived of such talents, of such services as their's!

But this is nothing to that which comes after. We all know the singular notion that has existed with reference to the inertness of French therapeutics—we all know how we hugged ourselves on being, at least, more active practitioners than our lively neighbours. This is another point on which we are corrected—this is another mental cataract that M. Baumés couches. The fact is, that the French have all along been the folks for active measures, and we have been the patrons of lavements, and ptisans, and eau sucré, and thin potages.

"Mr. Lawrence he mentions as an honourable exception to the general rule of ignorance and darkness in the progress of surgical science. 'I can affirm (he says), that I have seen him arrest a violent peritonitis, consequent on the operation for strangulated hernia, in as short a time as any French surgeon that has been accustomed to the rational therapeutical treatment of such affections, and this by his vigorous antiphlogistic remedies. *Had the patient been in any other hospital, I do not think he would have escaped death.*'"

The Italics are all our contemporary's own. He really believes this impudent Gascon—he seriously endeavours to persuade his readers that this nasty compound of unblushing ignorance, and equally unblushing lies, is a true picture of English hospital surgery. We have really no patience with the dolt that can retail such abominable trash. We would recommend the Editor to give his reviewer what, at Eton and at Westminster is technically known under the name of a *bummer*. It is the only way to teach him common decency and common sense.

---

AN ESSAY ON CLINICAL INSTRUCTION. By E. LOUIS, M.D. Physician to the Hôpital de la Pitié.

M. Louis divides the objects of clinical instruction and clinical study into two: first, into the study of particular facts, or of diseases considered in an isolated manner; secondly, into an inquiry into general facts. The lecture before us is much too long, and rather too abstruse, to enable us to go deeply into it at present, and we content ourselves with noticing merely that portion which relates to the real value of pathological anatomy.

"Another method of exploration," says M. Louis, "not less general, is Pathological Anatomy, which fixes the value of the others, and presupposes long habit and perseverance, in order to be a useful instrument in the hands of the practitioner. Pathological Anatomy declares the value of the other methods of exploration which it interprets. Without it, for instance, how should we know that crepitous *râle* indicates the first degree of pneumonia; that *gargouillement* and pectoriloquy demonstrate the existence of tuberculous cavities? that ægophony corresponds to an effusion of fluid into the cavity of the pleura? the symptoms of softening of the brain to that affection? those of adynamic fevers to a profound alteration of Peyer's glands? Evidently these facts would be unknown to us.

We might certainly, without the assistance of this science, and by the aid of the symptoms alone, determine the seat of a great number of maladies, but of their nature we should remain ignorant. The symptoms of apoplexy indicate sufficiently that the brain is the seat of this affection, it is the same with the symptoms of softening of this organ; but how, without the aid of pathological anatomy, could we know that the former are caused by a hæmorrhage, the latter by a softening or inflammation of the brain. But as the history of symptoms becomes valueless, unless they have been noted with great care, from their commencement to their termination; unless all the functions have been interrogated in the same manner: so pathological anatomy can only render to science all the service which might be expected from it, when we proceed with extreme care to an examination of all the organs, in those cases which proved fatal.

The object of pathological anatomy, in fact, is not merely to point out the seat of diseases, to show their nature, to verify or unravel their complications; it is the only means of arriving at the knowledge of a great number of the laws of the economy of disease which are the most important: and this knowledge can only be the result of an equally attentive examination of *all* the organs, in those cases where the patient has sunk under his disease, whatever it may have been.

If, for example, we know at this day that after the age of fifteen years, whenever we find tubercles or grey semi-transparent granulations in any organ, they co-exist at the same time, and in a still more advanced stage, in the lungs, it is to pathological anatomy, cultivated as I have just said, that we owe this fact. For if we had contented ourselves with examining in most cases the organs primitively and principally affected, evidently we could not have arrived at the knowledge of the law just mentioned, the importance of which cannot be doubted.

It is in the same manner, and by the same researches, that we have learnt that ulcerations of the pharynx, the œsophagus and the small intestines, are peculiar to two affections (I except syphilis) the one, acute, typhoid fever, the other chronic, phthisis, that ulcerations of the epiglottis, larynx and trachea are, with the exception already mentioned, peculiar to phthisical cases; that it is almost the same with fatty liver. And the importance of the laws announced by pathological anatomy, is still greater than at the first glance we might suppose; for these laws may in some instances, independently of the symptoms, in their absence, and even in opposition to their evidence, lead us to a rigorous diagnosis; thus chronic peritonitis, I mean that which is chronic at the commencement, is, in the adult, or rather after the age of fifteen years, according to all the facts which I have hitherto collected, constantly tubercular, or connected with the existence of grey semi-transparent granulations developed upon the peritoneum.

But, as I have previously mentioned, neither of these lesions exists in any organ, without appearing simultaneously in the lungs; so that if we have good evidence that chronic peritonitis exists, we may, independently of the symptoms derived from the respiratory organs, and even in their absence, announce the existence of a greater or less number of tubercles, or of grey, semi-transparent granulations, developed in the lungs. We may safely do so; for if observation should hereafter show any exception to this law, these exceptions will be rare, and will not affect the existence of the general law.

I have more than once recognized and announced the existence of phthisis in cases which exhibited all the symptoms of chronic peritonitis, but in which auscultation and percussion did not indicate any appreciable lesion of the parenchyma of the lungs; and even in individuals who did not cough:—a diagnosis which some persons will call presumptuous, which however was not so, and which I could not have failed to make without denying the laws of the economy of disease, the science itself in fact; for of what does the science consist but of these laws?"

None who practise the higher departments of our profession, none, in fact, who make it what it ought to be, a science, can fail to perceive the paramount importance of such great generalizations as the preceding. Such generalizations spring from an exact study of individual cases, and individual cases are themselves explained by the application of those generalizations—an example of the true spirit and the natural effects of inductive reasoning.

M. Louis takes up an important question, one too which has given rise to much dispute, and to some error. There can be no doubt that patients sometimes die, when dissection discloses no physical alteration of any consequence in any organ. Here the party, for such a party covertly or openly exists, opposed to the cultivation of pathological anatomy, imagine that the case makes against the utility of pathological investigations. Death, they say, takes place here without any great alteration of organs, and consequently when such alterations occur, they are not so important as they seem. M. Louis takes up the subject in a much more philosophical manner.

" But with whatever care we examine the state of the viscera, cases occasionally occur, in which the inspection of the body does not evidence any lesion capable of explaining either the fatal termination, or the symptoms observed; and it is cases of this sort, on which no doubt can be cast, which have given rise to the idea, that pathological anatomy has not all the importance which we attribute to it. But these cases are precisely those which prove in the most evident manner the indispensable necessity of this branch of the science, since if all the viscera had not been examined with scrupulous care, we could not have been certain that there was no serious lesion which would explain at once the symptoms, and the unfortunate termination of the case.

Whether then pathological anatomy shows, or does not show the reason of the phenomena observed during life, or the cause of the fatal termination of the malady, its utility is always the same; a contrary conclusion would in my opinion be opposed to evidence, and would declare that because some of the affections contained in our nosological chart are sometimes latent, the study of symptoms is useless.

We say, without hesitation, that pathological anatomy has neither been too much extolled, as some think, nor too much depreciated, as is the opinion of others; but it has been often ill understood. It is a method of exploration which no other can replace; that is all which can be said of it, and assuredly it is much to say."

This is indeed most true. Will the gentlemen who sneer at morbid anatomy find us any substitute for its exactness? Can they tell us how to read the language of symptoms? Will all the empiric observations of the Hippocratists, in every age, determine for us as much as of the nature, and the characters of disease, as the scalpel of the morbid anatomist has done? The answer to these interrogatories is obvious. Morbid anatomy is the only means of giving precision to diagnosis, an accurate direction to our treatment, because it is the only means of coupling symptoms with the states of the organs that occasion them.

The mode of connecting symptoms with lesions is thus dwelt upon by M. Louis.

" We must now ascertain the value of these symptoms, convert them into signs, as through them we are to arrive at a knowledge of the organ affected, or of the pathological state of the subject. For as I have previously mentioned at the commencement of this little work, there are cases in which the symptoms do not either at the commencement of the disease, or during an uncertain period of its progress, reveal any appreciable alteration of any organ whatever.

This second task, which constitutes in great part the art of diagnosis, offers no real difficulties in the majority of cases, at least to a man versed in the practical knowledge of disease; but in a considerable number of cases, these difficulties exist, and are not easily surmountable; and, in fact, are only to be overcome by considering the series and connexion of the symptoms; or by the assistance of the method of exclusion; that is to say, we arrive at the knowledge of the organ affected, not so much from the serious disturbance of its peculiar functions, as because, whilst the other viscera give no sign of any appreciable lesion, that alone ought to be considered as diseased, whose functions are in any degree altered, even though these alterations are not very characteristic: or again, because observation has demonstrated, because it results from well proven laws, that a certain alteration, or a certain evident disease supposes the existence of another still latent. Thus, as I have said above, chronic peritonitis supposes at once tubercles in the peritoneum and in the lungs; so that independently of the symptoms directly furnished by the respiratory organs, or in spite of the absence of such symptoms, we must conclude the existence of phthisis, from the single fact of the existence of peritonitis in a chronic form. If, in order to acquire an exact knowledge of any disease whatever, it is necessary to fix with precision the period of the appearance of the first symptoms, and to interrogate all the functions, this is especially indispensable in the cases of which we are now speaking; and it is in these circumstances also that we ought to make use of all the methods of exploration, especially of auscultation and percussion, for how many similar difficulties have been removed by these two methods! How many cases of pleurisy and pericarditis, for example, recognized by the employment of them, especially by percussion! Let us add, that to prove the accuracy of our diagnosis, it does not suffice to show, that the symptoms in the case under consideration, are those of this or that affection described by authors; it is also necessary to shew that the symptoms observed agree with a certain disease, and with no other.

But, having discovered the organ affected, it is necessary also to determine the disease of which it is the seat: a new, and in the present state of the science, sometimes an insurmountable difficulty; not so much from the divergence of the opinions of authors upon this point, as from the want of exact and numerous observation in a great number of diseases, from which information might have been derived; especially from the want of anatomical inspections carefully conducted at different periods of the same disease.

For it is principally by pathological anatomy, by the comparison of symptoms with the lesions which correspond to them, that a question of this sort can be resolved. Thus, when we wish to fix the seat of any affection, or to indicate its nature, we may, in the present state of the science, meet with insurmountable difficulties at the sick bed, and even after death.

These cases ought neither to be dissembled, nor removed from the observation of those who seek true instruction; for to dissemble the obscurities and difficulties of the science would be to deceive them. And besides, these cases are a valuable source of instruction, a means of strengthening the student in habits of observation, since we cannot decline to give a precise diagnosis in any case, until we have made use of all the means of investigation, compared with each other all the symptoms in their development and in their progress; until, consequently, we have recalled a great number of general facts, all of which ought to be incontestable: for to discuss upon doubtful or contested facts, would be, as it were, to fall willingly into error. These cases, then, are of all those which present themselves, the most capable of forming the judgment of those who apply themselves to observation; and of showing them, that the only way of making progress in the research of truth, is to admit as true, only that which is evident."

We now take our leave of this Essay on clinical instruction, which we recommend to every professor and to every student. It contains many sensible, though some perhaps are rather transcendental, remarks, on the best mode of studying and of pointing out the phenomena and laws of disease.

---

Dr. Carbutt's Report on Dropsy.*

Dr. Carbutt commences his report on this disease by remarks on the theory of absorption. He considers M. Magendie one of the most philosophical and accurate experimenters that ever lived. This eulogy is rather overstrained, at least we cannot consider M. Magendie as either very philosophical or very accurate; indeed the general opinion is opposed, and that very decidedly, to the accuracy of that gentleman. But let this pass.

Dr. Carbutt, from physiological considerations, concludes that in every instance of *passive* dropsy, the proximate cause is some impediment to the passage of blood along the veins, and consequently the non-absorption of the serous fluid. That obstruction, he observes, to the passage of blood along the veins is sufficient to produce dropsy, was shown by some experiments of Lower. He tied the vena cava inferior of a dog, which died in a few hours. Upon opening it, a collection of serum was found in its abdomen. The jugular veins were tied, when all the parts beyond them were found to be anasarcous, and not filled with extravasated blood, as had been anticipated. The impediment to the passage of blood along the veins may be some disease of the heart, as ossification of its valves, contraction of the orifice of the right ventricle, hypertrophy of the right ventricle. Or the impediment may be some tumor lying upon and pressing a great venous trunk, as an enlargement of the liver, spleen, or pancreas, pressing upon the inferior vena cava,—aneurism of the aorta,—ascites pressing upon the inferior vena cava and the iliac veins, and causing anasarca of the lower extremities,—pregnancy causing the gravid uterus to press upon the iliac veins, and also upon the inferior vena cava. Or, the impediment may be a torpid an indurated, or a scirrhous state of the liver, or, in short, any state of the liver which prevents or retards the circulation of blood through the vena portæ, which, as you well know, carries all the blood from the organs of digestion in tens of thousands of ramifications through the liver before that blood reaches the heart. Or the impediment may depend upon a plethoric state of the venous system, on account of the diminished secretion of urine, arising from some disease of the kidneys. Or, finally, the impediment may consist in the obliteration of one or more of the great venous trunks.

"When the impediment to venous absorption is some disease of the heart, as ossification of the valves, contraction of the orifice of the right ventricle, &c., our art is not able to do much. But, if the age and strength of the patient will permit, we may draw a little blood from the arm, thereby lessening the quantity which the heart will have to receive and transmit, and also encouraging absorption, which, as you have seen in M. Magendie's experiments, always takes place more rapidly when the quantity of blood in the circulating system is less. We may also administer digitalis partly to quiet the action of the heart, and partly to increase the flow of urine. We may administer mild purgatives, by way of carrying off a portion, at least, of the fluid which is continually entering, by the process of imbibition, into the intestines. We must also allow only mild and mucilaginous diet, in order partly to avoid exciting the heart, and partly to keep down plethora.—Lastly, when the hydrothorax, the ascites, or the anasarca, has increased to a certain degree, so as to be nearly unbearable, we may perform the operation of *paracentesis thoracis* or *paracentesis abdominis*, in the first two cases, or we may scarify or puncture the limbs in the case of anasarca.

When the impediment consists in an enlargement of any of the abdominal viscera, which press upon one or more of the venous trunks in the abdomen, as in many of the species of physconia, we must endeavour to cure the enlarged viscus; for which purpose the various preparations of iodine offer us the fairest chance. In the mean time we must palliate the dropsical symptoms as we

* Clinical lectures in the Manchester Royal Infirmary. Octavo.

best can. In the case of an aneurism of the aorta, I am afraid little or nothing can be done. In the case of pregnancy, you must bandage up the lower extremities, keep the bowels open, and wait patiently for a happy delivery. In the case of an ascites, which produces anasarca by pressing upon, and impeding the circulation in, the vena portæ, the vena cava inferior, the external iliacs, all I can say is, that you must endeavour to cure both kinds of dropsy as rapidly as you can. If you cure the ascites the cure of the anasarca will usually follow."

Dr. Carbutt does not mention the powerful effects of elaterium, powerful, according as they are directed, to good or evil. He points out the advantages derivable from the exhibition of small doses of mercury in enlargements of the liver, and the frequency with which alterations or enlargements of this viscus coexist with abdominal effusion. But the Doctor also has a secret to communicate.

"But, suppose the patient has taken as much mercury as the system will well bear, suppose the mouth is sore, and the old decayed teeth are dropping out, and the liver is still not restored to its healthy action; well, then I have a secret to tell you, or rather I told it you whilst we were treating Margaret Kearney. A secret, I call it, although it is really no secret; for I have told it every where. A respectable medical man should, indeed, have no secrets,—because he who knows of a means of doing good to his fellow-creatures, and does not disclose it, can be nothing but a genuine object of contempt. The secret, however, as I call it, is this, that an aloetic pill taken in a small dose frequently repeated through the day, has in a period of time, longer or shorter as the case may prove, the same effect in restoring the liver to a healthy action as is produced by mercury. This fact depends upon the principle which I have already explained to you, that irritation at one extremity of a canal in the living body excites action at the other extremity."

We should be sorry to express a doubt, yet we do doubt, if the aloetic pill will ultimately be found to have the same effect as mercury upon the liver. But we recommend our readers to put this to the test.

"As to obstruction of the passage of blood through the veins, arising from plethora,—that plethora depending on diminished secretion of urine,—I have to say that this generally depends upon disease of the kidneys; but if you expect that this disease of the kidneys will be infallibly pointed out by the albuminous state of the urine, you will be much disappointed. In this case you must, if the patient's strength will allow, apply cupping-glasses or leeches over the kidneys. You must administer diuretics, and if these fail, drastic purgatives, as elaterium, so that you may bring away the fluid by the intestines, and by any means lessen the excessive quantity of blood in the circulating system."

There is much truth in the observation that an albuminous condition of the urine is not an infallible evidence of disease of the kidneys. We have seen it on several occasions when dissection shewed the kidneys perfectly sound. At the same time the examination of the urine is desirable in all cases of dropsy, as indeed in a variety of other maladies. The following observations on obliteration of the venous trunks are not undeserving of attention.

"With regard to the obliteration of the venous trunks as a cause of *passive* dropsy, Dr. Bouillaud has, by numerous observations, fully proved its reality; and he has also showed that in a great number of cases, this obliteration was produced by sanguineous concretions or coagulable lymph effused in the body of the veins. He made these observations immediately after M. Magendie had published his new views of absorption.

Dr. Bouillaud reported three cases of dropsy of the lower extremities with obliteration of the principal veins of those members. He found also dropsy of the upper extremities when their principal veins were obliterated.

If the obliteration of the veins were confined to one member, then that member alone was affected with œdema; but if the obliteration took place in the veins of two members, whether upper or lower, then both those members were affected. If the obliteration took place in the inferior vena cava, then the dropsy was confined to the parts from which the inferior vena cava receives its branches; and when the superior vena cava was obliterated, then the dropsy affected only those parts of which the venous system empties itself into the superior vena cava. So that it became impossible not to admit that dropsies were occasioned by an obstacle to venous circulation, and were not the result of *debility*.*

Dr. Bouillaud also reported three cases of *passive* dropsy of the belly,—ascites,—in which there existed an obliteration of the vena portæ, that is to say, of the venous passage which performs the same part in the absorption and carrying away of the abdominal serum, as is performed by the brachial and femoral veins, and their coexistent veins, in the absorption and carrying away of the serum of the cellular membrane of the limbs.

Dr. Reynold has published† several cases of obliteration of the iliac veins, and of the inferior vena cava, in which there was manifested a dropsy of the lower extremities. He has also published three cases of ascites arising from obliteration of the vena portæ.

Dr. Tonnellé has published‡ six cases of obliteration of the venous sinuses of the dura mater, with serous effusion in the cavity of the arachnoidea.

With regard to the cure of *passive* dropsy arising from obliteration of the veins, I believe I may say it is entirely hopeless, any further than as Nature may choose to take it upon herself; and she certainly sometimes does wonders in this way. In the case of a woman, whose left iliac vein was obliterated, a very large vein coming from the femoral veins of the same side traversed the abdominal wall, making several turns, as high up as the umbilicus, it then bent itself in order to go down and enter the femoral vein of the other side. Single at its origin it divided itself into two or three branches, which soon reunited to form one trunk towards its termination. When the patient stood up, this vein was of an enormous volume, and equalled the size of the little finger. By the help of this anastomic circulation, the blood brought by the left femoral vein was carried almost entirely into the right femoral vein. At the same time as this anastomic venous circulation was established, a dropsy of the left inferior extremity became gradually dissipated. This is only a specimen of spontaneous cure; there are many other instances on record equally curious."

The second species of dropsy, or the *active* form, appears to have its seat in the arterial system, and to approach very nearly in its nature to inflammation of the part in which it is situated. Its character seems to lie about half way between the regular normal state of the secretions, and the secretion of inflammation. In the secretion of parts in their healthy state, no more is thrown out by the arterial branches than is taken up by the venous branches. In the secretion of inflammation there is thrown out a quantity of coagulable lymph, consisting, Dr. C. thinks, almost entirely of fibrine.

"The symptoms of *passive* dropsies are slowly developed. They are for the most part the result of the compression which the serous collection exerts as a foreign body upon the surrounding parts. The symptoms of *active* dropsies make on the contrary a very rapid advance, and, in this kind, besides the phenomena resulting from the pressure of the effused fluid, we observe a variety of symptoms which denote an increase of vital power and action.

With respect to the causes of *passive* dropsy, I have already said nearly enough; but I omitted to mention that one of the causes of obliteration or obstruction of the veins is *phlebitis*, or inflammation of a vein, and that it produces its effect by causing sanguineous

---

* J. Bouillaud. Art. Hydropisie.—Dict. de Med. Tome Xme.

† Journal Hebdomadaire de Médecine et de Chirurgie.

‡ Ibid. Tome v.

concretions or effusion of fibrine in the trunks of the veins. As to the causes of *active* dropsies, a sanguine or plethoric constitution, with the use of such articles of diet as are likely to produce the plethoric state, are to be reckoned among the predisposing causes. With regard to the occasional causes, the most frequent is the operation of cold and damp, especially when suddenly applied to a person in a state of perspiration, whether in consequence of violent exercise, or of the prolonged heat of the weather. Stout men, after forced marches, exposed to causes capable of abruptly suppressing perspiration, fall suddenly into the *active* kind of dropsy. Soldiers fording rivers, whilst in a state of perspiration, are particularly liable to it."

The following is the Doctor's treatment.

" In *active* dropsy, if the patient be young and plethoric, you must take blood from the arm. You must apply leeches locally. You must use the warm bath, blisters, diaphoretics, purgatives, and energetic diuretics. You must also employ mercurial preparations, as the action of mercury upon the system, has the same power in removing the hydro-phlegmasia, or *active* dropsy of serous or cellular membrane, as I have already intimated to you that it possesses in removing inflammation of those membranes. Above all things, the diet must be the lowest possible."

We do not observe much to detain us in Dr. Carbutt's remarks on anasarca. When general dropsy commences with anasarca of the lower limbs, this is, cæteris paribus, presumptive evidence of the cause of the dropsy residing in the heart. This is a general fact of some importance, on which Dr. Carbutt lays insufficient stress. Active anasarca frequently attacks individuals, who, when convalescent from eruptive fevers, expose themselves to a cold and damp atmosphere. As to the prognosis, says our author, of *active* anasarca, it is good; of *passive* anasarca, without ascites, it is bad: of *passive* anasarca with ascites, and without any disease of the heart, it is not so bad.

"The mode of treating anasarca does not differ from that of treating dropsy in general; with this difference, however, that punctures and scarifications may be employed to give exit to the serum, and compressive bandages to support the tumid limb. We must recollect, however, that the incisions sometimes give rise to ulcers, which will not heal, to erysipelatous inflammations, and even to true gangrene. The making of punctures with sharp needles is the best way of avoiding these unpleasant consequences; but sometimes it is necessary to run all risks for the sake of immediate relief."

We extract the preceding passage, in order to point out, as forcibly as possible, the propriety of puncturing anasarcous parts with a needle, in preference to a larger instrument. True it is, that even such punctures as these *sometimes* give rise to erysipelatous or diffuse inflammation. Yet this is usually not the case; and, at all events, it much less frequently occurs as a consequence of small needle punctures than of larger ones.

" In the *hydrothorax*, hydrops thoracis, or dropsy of the chest, the affection sometimes arises from hyper-secretion of the pleura, which constitutes *active* hydrothorax; and sometimes it arises from a diminution of venous absorption, which constitutes *passive* hydrothorax.

*Active* hydrothorax is not easily distinguishable from a slight acute pleurisy; and frequently a chronic pleurisy leaves behind it a serous effusion, which differs in no respect from hydrothorax not preceded by inflammation.

Hydrothorax ordinarily occupies only one side of the chest. The quantity of the effused fluid is very variable; it has reached twelve pounds. The lung corresponding to the effusion is compressed, and thrust towards the vertebral column and the top of the thorax, whilst the diaphragm, with the liver or the spleen, according to the side affected, are pushed downwards. When the effusion occupies the left side of the chest, if it is very abundant, it throws the heart towards the right side.

The causes of hydrothorax are those of dropsy in general. So that *active*

hydrothorax may be the consequence of a sudden suppression of the perspiration, or of some other secretion. *Passive* hydrothorax is met with in persons affected with a disease of the heart or of the large vessels, capable of opposing an impediment to the free circulation of the blood."

On the symptoms of hydrothorax we need not touch. Suffice it to observe, that it is very seldom a substantive complaint, unattended with some serious organic alteration, commonly of the heart or the great arteries. But, as Dr. Carbutt properly remarks, active hydrothorax is more frequently a complaint in itself than the passive form. The old ideas on the subject of hydrothorax are, we need not say, preposterous.

On ascites, we see nothing but the following observations to detain us.

" The *occasional* causes of *active* ascites are the same as those already mentioned in speaking of anasarca and hydrothorax. As for *passive* ascites, it used to be the fashion, and it no doubt is still the fashion, to assign a great number of occasional causes, which were considered sufficient to *debilitate* the lymphatic vessels, as the *atony* of those vessels, or their *debility*, was considered as the *proximate* cause of the disease. But it is time to have done with so unmeaning an hypothesis as *debility*, when we see patients die daily of debility without any symptoms of ascites, or of any other species of dropsy: as in consumptions, gastro-enteritis, fevers, in which there is sufficient debility, but with an evident increase instead of a deficiency of absorption. The *proximate* cause of *passive* ascites is, like the proximate cause of *passive* general dropsy, the non-absorbing of the veins; the cause of this is some interruption to the circulation of blood along the veins; the cause of this I have already dwelt upon."

We are disposed to go to some extent with Dr. Carbutt, in repudiating the idea of mere debility doing so much mischief as it enjoys the credit of effecting. There is probably, in *most* cases, some efficient cause, which plays an important and immediate part in the production of dropsical effusion. Whether this is so constantly to be looked for, or found in the veins, as Dr. Carbutt appears to believe, may, we think, admit of question. Yet the agency of general debility must not be overlooked. *That,* perhaps, occasions many functional and structural organic changes, which themselves give rise to dropsical effusions.

Dr. Carbutt presents ten cases of dropsy, mostly of a passive character, and affecting the abdomen and the limbs. We shall notice only one, for a particular point which it illustrates.

CASE 1.—*Passive Dropsy of the Belly and the Limbs.*

July 1, 1833. Margaret Kearney, aged 46, married, takes care of her house, has been ill for four months. Tongue is foul. Has a bad taste in her mouth. Bowels costive. Voids a very small quantity of urine. Belly is swelled, with a distinct fluctuation. There is pain over the hepatic region, and the liver seems enlarged. The legs and feet are swelled, and pit upon pressure. She has some difficulty in breathing, and a short cough. Pulse is rapid, small, and weak. No catamenia for four months past.

Dr. Carbutt put the patient upon digitalis, with occasional doses of castor oil. But the symptoms became worse rather than better—the stools continued clay-coloured—and the digitalis depressed the heart's action. On the 10th, it was omitted, and one grain of calomel night and morning, with two ounces of gin daily, were substituted for it. On the 13th the mouth was sore. The stools were still clay-coloured, and there was tenderness in the hepatic region. On the 16th, these symptoms were undiminished, and four ounces of blood were abstracted from the region of the liver by cupping. This measure greatly diminished the pain. On the 17th, the swelling was less, the flow of urine greater—the stools unchanged in character. The mouth was so sore, that two teeth had dropped out.

To omit the calomel pills.

To continue the gin.

To take two grains of the Pilula Aloës cum Myrrhâ every hour whilst waking.

The bowels were well acted on by the aloetic pills, and on the 21st, the stools were more natural. On the 24th she was ordered a draught of infusion of calumba with carbonate of potass. On the 28th, the gin was increased to 4 ozs. daily. On the 31st, the stools were more bilious. On the 1st August, she was ordered a morphia draught, on account of want of sleeep. On the 8th, the stools had become quite natural. We need not pursue the diurnal reports, for on the 19th, the patient was discharged cured.

We have taken the preceding case because it illustrates Dr. Carbutt's opinion respecting the action of aloes on the liver. We do not mean to deny to aloetic, what perhaps should be conceded to other purgatives, that directly or indirectly they may influence the liver in some slight degree; but, without appearing hypercritical we may observe, that in this instance the evidence is not so conclusive as to carry demonstration on its face. For, the biliary secretion was long in being established, and the post hoc is more certain from many considerations than the propter hoc. Dr. Carbutt, however, feels no doubt upon the subject, and it is fair to allow him to speak for himself.

"This, gentlemen," he says, or rather said, "was a case of passive dropsy from obstruction of the liver. As soon as we procured bilious stools, the urine became more plentiful, and the swellings disappeared. You find it reported towards the conclusion of the case that the urine became more scanty; but I apprehend that this remark originated in a slight mistake. As she was taking a small aloes pill every hour whilst waking, it is most likely that she voided a great part of her urine with her stools, which were numerous, and that therefore the quantity of her urine was not fully estimated. In this case you have seen put into practice the secret of which I told you in my lecture upon dropsy in general. You saw that this woman took calomel until her mouth was very sore, and two unsound teeth dropped out; yet her stools continued clay-coloured. I omitted the calomel, and gave her a small aloetic pill every hour whilst waking; knowing that, according to theory, the passing of this pill so frequently over the mouth of the ductus communis choledochus, must sooner or later rouse the liver to action, and consequently to the regular secretion of bile. For a week or or two I was disappointed; but I still persevered, saying that the plan was rational. When our plans are rational, we ought to persevere: we cannot always command success; we must sometimes be content if we deserve it. At last the wished-for bile appeared; at which I honestly confess I felt more delight than I believe a sportsman does upon finding his game. 'Here is the triumph of theory!' I said to Mr. Lloyd. 'Sir,' replied he, 'I think it is the triumph of practice.' He was right; and we were both right. Good theory, and good practice, are one and indivisible. They are in fact the same thing. As long as you practise, gentlemen, always consider the why, and the wherefore. Ask yourselves what you intend to accomplish, what is your object, and then meditate most earnestly on what may be the rational mode of attaining that object. You had better, however, keep your theories to yourself, unless, like me at this moment, you have a public duty to perform."

We do not perceive any peculiar interest in the other cases. Yet they well deserve the attention of those, who are anxious, as all should be, to study facts. We therefore refer our readers to the volume in which they are contained.

---

### Lock Hospital.

### Some Cases of Venereal Affections of the Fœtus and of Children. By H. J. Johnson, Esq.

The following brief memoranda of some cases may not be totally undeserving of attention. I have few observations to offer on them, and would merely recommend the subject of syphilitic affections communicated from the mother to her offspring, to the more deliberate consideration of the profession, than it appears to have hitherto received.

*Case* 1. A strong looking Irish-woman presented herself at the hospital, and brought with her her child then nearly three weeks old. She stated that she had been a patient in the hospital four or five years before, on account of a syphilitic complaint communicated to her by her husband. She was then pregnant. The syphilitic symptoms disappeared under the treatment employed, and she quitted the hospital, apparently cured. Soon after her dismissal, she was brought to bed of a still-born child, nearly at the full time. The fœtus was covered with blotches on the skin.

From that time the mother had had no venereal symptoms whatever, either of a primary or secondary character. She had become pregnant four times subsequently to the accouchement I have mentioned. On the two first occasions, the child was born dead, nearly at the full period, and each time it was covered with cutaneous blotches. The third pregnancy ended in the birth of a living child. It was born without any syphilitic symptoms, but in three weeks or thereabouts after birth, an eruption made its appearance, the child drooped rapidly, and soon died.

The fourth child was the one she brought with her to the hospital. It was very small and delicate, had slight ophthalmia neonatorum, but displayed no syphilitic taint. The mother was strong and apparently healthy, and, as I have already stated, she had had no secondary nor primary symptom for five years or upwards. She declared that she was confident the child would die as the last child had died.

In a month after this visit she returned to inform us that the child was dead. In a few days after she left the hospital an eruption, of a brown colour and a scaly character, appeared upon the infant; it emaciated with rapidity, and it shortly sank.

It is certainly a curious circumstance that successive infants should be thus affected by the syphilitic poison which would seem to have completely left the mother. The ova must have been poisoned. Perhaps the case of the mare that was covered by the quagga was analogous, in some respects, to this—analogous, at least, in the fact of a temporary influence exerting an effect on more ova than those that were impregnated at the time that influence was exercised. A mare was covered by a quagga. The foal was striped, as might naturally have been anticipated. Subsequently the mare was covered by a stallion, and the foal again had the quagga stripe.

In the case of the woman I have mentioned, the influence on the ovum indeed was somewhat different. For in her it was not the influence of imagination, which might have done something in the instance of the mare; but the syphilitic poison, probably affected ova, which in after years were to be impregnated. At all events the facts are worth recording.

*Case* 2. When residing at the hospital as house-surgeon, an out-patient brought her child for my inspection. She had been a patient in the hospital 12 months previously, on account of a sore upon the nympha. She was pregnant at the time. After remaining for two months in the hospital, and undergoing a course of mercury she was discharged, apparently quite well. Five months after her dismissal she was confined. The child, a male, was healthy at the time of birth, and for five months after birth remained so. He then was affected with an eruption on the skin, for which the mother brought him to me as I have stated.

The eruption consisted of spots of what approaches nearer to the characters of the psoriasis guttata, than of any eruption sufficiently familiar to the bulk of the profession, to admit of being selected as an object of comparison. It was very general upon the trunk, not much diffused upon the limbs. The child's health was tolerably good, he was plump, and had no other symptom of a syphilitic character. The nature of the eruption admitted of no doubt; it was unequivocally venereal.

I ordered some powders containing the hydrargyrus c̄ cretâ for the child, and prescribed sarsaparilla for the mother. The child, of course, was suckling, and I presume there can be no doubt of the advantage of giving the mercury to the infant in preference to the parent. For, if the mother takes it, two persons are subjected to its influence instead of one, a gratuitous injury—and, besides, the infant receives the mineral through the medium of the milk, its food, which is so far poisoned, instead of being the vehicle of wholesome nourishment. On the other hand, when mercury is given to the infant, and the mother is treated with sarsaparilla or with other tonics, her health is supported, and she offers to her child, what it surely must require, a supply of sound and generous nutriment.*

In the present case, the remedies so exhibited answered well. The eruption desquamated, faded, and disappeared—the child's health continued good—and the mother's was rather improved than otherwise. I continued the hydrargyrus c̄ cretâ for a month, indeed for rather more, and the mother persevered with the sarparilla for a longer time than that.

For two or three months after this occurrence I saw nothing of the parties. At the expiration of that period, the woman returned for my advice. The child was much emaciated, and at the verge of the anus on the right side was an ulcer, nearly as large as a sixpenny-piece. The base was indurated, the edges irregular and hard, the surface yellowish, the surrounding skin inflamed. In the left groin was a bubo, as large as a pigeon's egg, inflamed, and tender. The child was feverish, the secretions depraved.

I ordered salines, mild purgatives, and the warm bath. Under these means the local inflammation and the general febrile symptoms subsided. I then directed the mother to take the sarsaparilla combined with very small doses of the oxymuriate of mercury. The child gradually improved—the ulcer healed, with hardness of the cicatrix—the bubo passed away—the induration of the cicatrix disappeared, and the child very slowly recovered flesh and strength. I saw it two or three months ago. It had small-pox, subsequent to vaccination, very severely in the interim. But it was now grown strong and plump, and no venereal affection was apparent.

It is singular that in the preceding case the child, which must necessarily have been infected in utero, should be born quite healthy, and remain so for five months after birth. It then displayed such secondary symptoms as are not unfrequent. I need say no more upon the mode of treatment, but perhaps I should remark, that when the ulceration near the anus and bubo made their appearance, the child was so debilitated and reduced, that there seemed little prospect of a permanent recovery.

One other observation, desultory as it is, may be permitted. It is not very uncommon for a patient to present some mild form of venereal eruption, psoriasis for instance, or lepra—to get rid of this under proper treatment—and soon afterwards to be affected with a worse kind of eruption, rupia perhaps, or cachectic ulcerations. If the patient is treated violently with mercury, or much reduced in any way during the continuance of the first eruption, this is frequent enough, and may be readily accounted for; but I mean to affirm, that where the patient is *not* reduced, where mercury is properly and mildly given, the circumstance alluded to occasionally occurs. Yet, so far as I have seen, rupia, or ecthyma, or cachectic ulcers so occurring, are much more tractable than where they follow the abuse of mercury, or some obvious debilitating cause.

*Case* 3. In March of the present year, I was requested to see the child of the wife of an opulent tradesman. The child was eleven months of age.

At the commissure of the penis and scrotum was a small yellowish brown

---

* Of course this reasoning is inapplicable to cases, in which the mother is labouring *at the time* under syphilitic symptoms. Then to treat her, is to treat the child also.

spot, with some thickening of the cutis, and a disposition to the formation of a pustule. A similar spot was present on the inferior surface of the scrotum. The tonsils were rather larger than they should be. The child was delicate in its appearance, but its health was good.

The following is the history of the case of the mother and child. In 1833, the mother had been infected by her husband, and suffered from a primary sore of the labium. For this she took mercury, and was under its influence for nearly five months. During the last month of this time the extremity of the nose had been the seat of and indeed was destroyed by an ulcer, which commenced as a pustule with an inflamed areola. It was at this period and in this condition that I first saw the patient. I ordered sarsaparilla in full doses—the ulceration of the nose healed up, without attacking the nasal bones—and the patient was to all appearance cured. Three or four months after this I again saw her. This was in September 1833. She had then an eruption of brownish red spots, inclined to vesication, and she had also slight ulceration of the nymphæ, which had evidently originated in a spot of secondary ulceration. I again ordered the patient sarsaparilla combined with occasional aperients. The eruption lasted more or less till June 1834. From that time to the present, upwards of twelve months, the mother has continued well, and is now in robust health. When she first came under my observation she appeared in a very precarious condition.

In April, 1834, the mother, then suffering under the eruption already mentioned, was confined and gave birth to a male child. At birth it was to all appearance healthy, but in ten days afterwards a "scabb'd" eruption occurred upon the face, and "red spots" covered the body. I had not an opportunity of seeing the child at this period; it was treated by another surgeon. He ordered "blue powders," which the child took every night for three months. The eruption lasted two months from the time of its commencement. The child continued well until it was six months old, when another eruption occurred of "red spots," attended with general loss of the nails. This lasted for six weeks, during which the child was taking a "white powder," under the direction of the surgeon who had treated it before.

The two spots for which the child was now brought to me, had lasted for two weeks, and were increasing in dimensions.

I now prescribed.

*Hyd. c̄ cret.* gr. i. *Pulv. cret. comp.* gr. iij. *Omni nocte, pro infante,*

and gave the mother some sarsaparilla; she was still suckling the child. The spots on the latter disappeared in about a fortnight, and all stain disappeared also. The health of both mother and child was good, and after about a month, I discontinued all medicines for both. I saw the child a few days ago, and it continued free from all venereal symptoms.

These cases are offered with few or no comments. They are the simple statements of facts comparatively unconnected. Perhaps they tend to shew the influence of treatment on the secondary syphilitic eruptions of infants; and perhaps they also tend to illustrate the advantage of maintaining as good a supply of healthy milk as possible from the mother to the child, while the latter is undergoing the remedies adapted to the specific symptoms it displays.

---

### ST. GEORGE'S HOSPITAL.

In the last Number but one of this Journal, we seized the opportunity of offering a few remarks on the principle of appointing several assistant surgeons to hospitals. We shewed, or at least we endeavoured to shew, that such a principle is founded in liberality, expediency, and justice: and that the limitation of the number is exactly in the ratio of the limit laid down, an approach to the spirit of monopoly. The object of those who administer the affairs of our medical charities should be, to train up efficient medical officers, by holding out encouragement, and a prospect of reward, to the most industrious and most talented young men. In a practical science, like that of medicine, physicians and surgeons are not to be obtained ready-made, of any quality. They must serve a long apprenticeship of exercise and study, not the study of books, nor even of lectures, but of the human body. They must early have the opportunity of treating cases, and of acquiring the tact that long-continued practice only can confer. It is on this account that so many of our military and naval surgeons have succeeded in civil practice to a remarkable degree, although they have "set up" late in life—perhaps with little interest, and with many disadvantages. It is on this account, too, that many gentlemen, who have lived in London, and practised privately, have failed as hospital surgeons and physicians, when those appointments have not been obtained at an early period of their career.

There cannot be a doubt in the mind of any unprejudiced person, that the opportunities afforded to young men, for gradually acquiring the habit of *treating* disease, should be abundant—as abundant as by possibility they can be made. They encourage industry, give a field to exertion, exhibit talent, and rear a class of practical men, for the benefit, not only of the poor but of the rich. The matter will not bear questioning for one moment—the point is as clear as that the sides of an equilateral triangle are equal. There is nothing to be urged against it but private interest; and, at this time of day, such an argument is most dangerous to the man that wields it. We, therefore, advocated the appointment of two assistant surgeons to St. George's Hospital, in lieu of only one—the governors of the hospital took the same liberal view of the case—and the public, the medical public we mean, must rejoice in this triumph of the utilitarian principle:—the greatest good to the greatest number.

As governors of the institution, we have been lately summoned to its board-room, to take into consideration a code of laws, for the regulation of the charity, proposed by a committee formally appointed. We observe much discussion on the subject of these laws in some of our weekly contemporaries. The Lancet has sharply attacked the laws, as well as the committee from which they emanated. Having looked at the matter with as much deliberation and as much judgement as we are capable of exercising, we are disposed to think that the Lancet has been misinformed, both as to the nature and the tendency of those laws. We really can discover no leaning on the part of those who framed them, nor any obvious bias in the laws themselves, towards the interests of the medical officers of the hospital. On the contrary they appeared and still appear to us to be well calculated, on the whole, to promote the well-being, and good management of the institution. We have therefore at some personal trouble supported them by our private votes, and we have no objection to give them our support also as public journalists. Setting aside prejudice, and some degree of clamour, we really and conscientiously think that the laws in question are extremely good. There is one fault perceptible enough in their composition—a trivial one perhaps, yet one which might be advantageously amended—we allude to the occasional difficulty experienced on the part of the puzzled reader, in distinguishing the language in which they have been written. English it certainly is not, at least sundry sentences display an independent spirit of diction which sets at defiance the cramped and sullen rules of Murray.

But our readers can take little real interest in the affairs of St. George's Hospital, except when they bear upon some question of general policy or usefulness. The only one to which it is at present worth while to allude, is a law which was proposed, and which indeed has been almost unanimously carried, to admit of the expulsion of any governor proved to be unworthy.

It has been urged that it is dangerous to give the governors the power of expulsion from amongst them, of any member of their body. But surely this is a monstrous doctrine. There is not a pot-house club in the kingdom—there is scarcely a respectable or aristocratic association, whether for the purposes of usefulness or pleasure, which has not the right of expelling one who injures or disgraces it. And is St. George's Hospital to be destitute of this wholesome and salutary power —is it to be debarred from excluding from its board-room a scoundrel or a felon? The idea is too preposterous to require refutation. Any body of men that enjoy the right of election of their members, should also possess the power of expulsion; that is clear in point of principle, and almost universally acted on in practice. The Governors of St. George's Hospital cannot be blamed for demanding a power of which they should never have been deprived.

No one then can deny the justice of a public body being invested with the privilege of turning out an improper member. The great point is to guard against the possibility of abuse—to prevent any party from making use of this common weapon for their private purposes. The great check against an abuse of this description is publicity. The whole process of expulsion from first to last should be as public as it possibly can be. It has been urged against publicity that it is not mercy to the accused or guilty party. Mercy, however, must yield to justice. The great object is to guard against the abuse of power, to prevent parties from crushing individuals. It is more likely that such abuse should be perpetrated under the protection of privacy, than that innocent persons should suffer from publicity, or that public morality should be shocked by it. The advantages of publicity more than counteract its evils. This is the great principle of modern legislation.

In the first instance, it was proposed by the Committee that the Weekly Board should possess the power of expelling a governor for obvious misconduct. This expulsion must be confirmed at the ensuing weekly Board, and the individual implicated had the power of appeal to the General Quarterly Court. This proceeding was adopted to avoid extreme publicity, yet to offer, at the same time, the advantages of a double appeal to the accused. To this law the Lancet directed the indignant attention of the public. We think the anger and indignation of our contemporary were overstrained; we think there was no necessity nor foundation for making the law the subject of a direct attack on the Committee; but, we also think that the law was not a good one, that the mode of expulsion was not attended with sufficient publicity, nor guarded so strictly as it ought to be against abuse.

We were therefore inclined to oppose this law, when we found, with much pleasure that the medical officers took this view of the question themselves, and brought forward a distinct proposition to permit the expulsion of a governor of the Institution at a Special Court, and a Special Court only. That governor might previously resign if he thought fit—a humane provision to him, and a proper one for the purpose of avoiding scandal to the public.

Thus the matter has been settled to the satisfaction of every liberal and independent person. We must say that we consider the conduct of the medical officers of the Institution candid, and gentlemanly in every respect, and we, as governors, in this instance perfectly unprejudiced, see no ground for dissatisfaction at the course they have pursued. Nay, we fancy that the Editor of the Lancet, on consideration, will perceive that he has been led away throughout the whole discussion, by persons who have been misinformed with respect to the state of affairs at the Hospital.

